# Epigenetics and Communication Mechanisms in Microglia Activation with a View on Technological Approaches

**DOI:** 10.3390/biom11020306

**Published:** 2021-02-18

**Authors:** Sabrina Petralla, Francesca De Chirico, Andrea Miti, Ottavia Tartagni, Francesca Massenzio, Eleonora Poeta, Marco Virgili, Giampaolo Zuccheri, Barbara Monti

**Affiliations:** 1 Department of Pharmacy and Biotechnology, University of Bologna, 40126 Bologna, Italy; sabrina.petralla2@unibo.it (S.P.); francesca.dechirico3@unibo.it (F.D.C.); andrea.miti@unibo.it (A.M.); ottavia.tartagni3@unibo.it (O.T.); francesca.massenzio2@unibo.it (F.M.); eleonora.poeta3@unibo.it (E.P.); marco.virgili@unibo.it (M.V.); 2 Interdepartmental Center for Industrial Research on Health Science and Technologies, University of Bologna, 40126 Bologna, Italy; 3 S3 Center of the Institute of Nanoscience of the Italian National Research Council (CNR), 41125 Modena, Italy

**Keywords:** neuron–microglia crosstalk, epigenetics, exosomes, miRNAs, microfluidics

## Abstract

Microglial cells, the immune cells of the central nervous system (CNS), play a crucial role for the proper brain development and function and in CNS homeostasis. While in physiological conditions, microglia continuously check the state of brain parenchyma, in pathological conditions, microglia can show different activated phenotypes: In the early phases, microglia acquire the M2 phenotype, increasing phagocytosis and releasing neurotrophic and neuroprotective factors. In advanced phases, they acquire the M1 phenotype, becoming neurotoxic and contributing to neurodegeneration. Underlying this phenotypic change, there is a switch in the expression of specific microglial genes, in turn modulated by epigenetic changes, such as DNA methylation, histones post-translational modifications and activity of miRNAs. New roles are attributed to microglial cells, including specific communication with neurons, both through direct cell–cell contact and by release of many different molecules, either directly or indirectly, through extracellular vesicles. In this review, recent findings on the bidirectional interaction between neurons and microglia, in both physiological and pathological conditions, are highlighted, with a focus on the complex field of microglia immunomodulation through epigenetic mechanisms and/or released factors. In addition, advanced technologies used to study these mechanisms, such as microfluidic, 3D culture and in vivo imaging, are presented.

## 1. Role of Microglia in CNS Physiology and Pathology

Microglial cells, described for the first time by Pio del Rio-Hortega (reviewed by Kettenmann [1]), are the immune cells of the Central Nervous System (CNS) with phagocytic activity able to maintain the overall health of the brain microenvironment. Previously considered as being derived from blood monocytes (circulating precursors of macrophages), microglia, in the early 1990s, were suggested to originate from the embryonic yolk sac by Cuadros and collaborators [2]: This was later confirmed by Ginhoux [3]. However, the origin of microglia was not the only matter of debate about this cell type; even before its discovery, pathologists had identified microglial cells with a clearly different morphology in a healthy brain from those in a damaged one, as observed in 1878, by Carl Frommann, in a multiple sclerosis patient [1]. Moving forward in time and after research advances, the microglia plasticity and the related concept around the M2/M1 polarization are now widely accepted. Microglia are constantly screening the CNS environment, and, once activated, these cells can acquire a specific phenotype, according to the perceived stimuli. Since microglia have been defined as the macrophages of the brain, the classification and phenotype has been established based on macrophagic characteristic. An oversimplified and generally accepted classification suggests the classical activation or pro-inflammatory phenotype (M1-phenotype) in response to infection or tissue injury [4] and the alternative activation or anti-inflammatory phenotype (M2-phenotype) in response to neurodegenerative diseases [5] and in the context of primary and metastatic tumors [6,7]. Moreover, the mechanism of activation of the M2 phenotype is more complex than the M1, leading to a further classification in M2 subtypes. Treatment with interleukin-4 (IL-4) and/or interleukin-13 (IL-13) has been observed to induce the M2a phenotype, which is involved in tissues repair and growth stimulation. M2b phenotype is a mixed activation state that responds to IL-1β and LPS responsible for the production of TNFα and IL-6. Finally, an additional subtype of M2 microglia is the M2c phenotype promoted by IL-10 stimulation. Even if the M1 pro-inflammatory phenotype and the M2 anti-inflammatory one have been accepted by the scientific community, this classification is largely oversimplified, since epigenetic modulations can contribute to the different microglial phenotypes. To this aim, epigenetic modifications, such as DNA methylation, histones post-translational modification and non-coding RNAs (ncRNAs), are gaining more and more importance in modulating microglia biology and plasticity.

## 2. Neuron–Microglia Crosstalk in CNS Physiology and Pathology

Several evidences confirm the important role of the bidirectional communication between the nervous and the immune system in CNS in both physiological and pathological conditions. Indeed, the dynamic interactions between microglia and neurons influence the maintenance of homeostasis and the proper neuronal functioning of the healthy brain. More specifically, neuron–microglia communication is involved in neuronal development, synaptic plasticity, regulation of angiogenesis and programmed cell death besides immune response [8]. In pathological states, the bidirectional dialogue perturbation promotes microglia activation and the consequent increased inflammatory processes which in turn contribute to altering synaptic functions, plasticity and cognitive deficits, responsible for multiple brain pathologies, such as multiple sclerosis (MS), Alzheimer’s disease (AD) and Parkinson’s disease (PD).

A physical and direct connection of microglial cells with neuronal elements has been extensively demonstrated in both physiological and pathological conditions. In particular, in vivo studies have shown that microglia contact with synaptic structures preferentially localize at smaller dendritic spines, thus surveying excitability, quantity and quality of synapses [9,10]. Using two-photon imaging, the authors noted direct and repeated contacts between microglial processes and dendritic spines. Furthermore, in the developing brain, these microglia-to-neuronal spine contacts are prolonged, with morphological modifications of such spines induced by microglia during postnatal weeks. The growth in dendritic spines is due to the activation of CX3CR1-CX3CL1 or TREM2 pathway or to the release of interleukin -10 (IL-10) in the microenvironment, as confirmed by several studies, especially on the hippocampus [11,12].

Not only physical contact, but also the release of soluble factors contributes to neuron–microglia crosstalk both in physiological and pathological conditions. Physiologically, microglia–neuron interactions are crucial for an accurate brain formation during the prenatal and postnatal periods, but also in the maintenance of homeostasis in the adult stage, in particular by modulating neurogenesis, synaptogenesis and programmed cell death [11]. With reference to neurogenesis, the inflammatory molecules produced and released by microglia act on the microenvironment that controls the neural stem cells survival: In fact, TNFα signaling could improve or reduce neurogenesis by acting on TNFR2 or TNFR1, respectively. In the ventricular and sub-ventricular zone, microglia modulate proliferation, differentiation and maturation of brain precursor cells through phagocytosis [13]. In more detail, phagocytosis can be activated by pro-inflammatory stimuli through the recognition of pathogens via TLRs, or anti-inflammatory ones promoted by the recognition of apoptotic cell debris. Moreover, phagocytosis is stimulated by fibrinogen, metalloproteinases and cathepsins allowing microglia to act on synaptic remodeling [13,14]. Microglia take part in synaptic refinement, also by producing a variety of neurotrophic factors, cytokines and chemokines that are involved in neuronal survival allowing mature neurons to take part in neuronal networks. For example, CX3CL1-CX3CR1 and CD200-CD200R interactions contribute to preserve the microglial surveying state in neuronal activity and synaptic plasticity processes [15]. Thus, microglial cells are involved in the pruning of synapses, an activity-dependent process required for the maturation of neuronal circuits, especially during the postnatal development [15]. Going through the involvement of microglia in synaptic functions, it has also been demonstrated that the increased expression of complement proteins C1q and C3 promotes the engulfment and degradation of synaptic material through the interaction with the microglia-specific C3 receptor (CR3). Furthermore, microglia can induce programmed neuronal death, thus contributing to balance neuronal number from overproduction during development, as well as synapsis number, through the removal of damaged or inactive synaptic connections [16]. Defects in microglial control of these physiological processes can contribute to neurodevelopmental disorders and neurodegenerative diseases. In AD, as well as in PD, epilepsy and MS, cognitive disability may be related to uncontrolled synapse pruning, whose defective neuronal architecture can be caused by excessive microglial phagocytosis [13].

**Figure 1:**
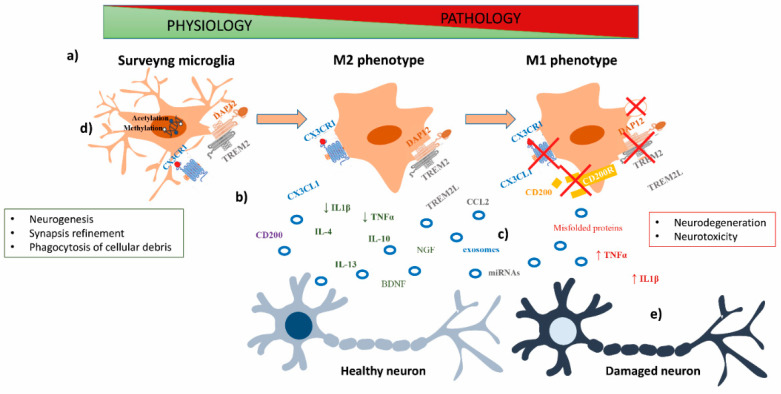
Communication mechanisms in neuron–microglia crosstalk. (**a**) Microglia activation after different stimuli can culminate in a pro-inflammatory or anti-inflammatory activation, consisting of morphological and biochemical modification. (**b**) To exert biological functions, microglial cells adopt physical and direct contact of microglial cells with neuronal elements; they also release soluble factors (**c**) and exchange biomolecules through secreted vesicles, contributing to neuron–microglia communication in both physiological and pathological conditions. These molecules include neurotrophins, cytokines (as ILs and TNFα), chemokines (as CCL2 and CX3CL1) and nucleic acids (e.g., miRNAs). (**d**) Additionally, neuron–microglia crosstalk can be mediated by epigenetic reprogramming included changes in histone acetylation and methylation. (**e**) Defects in microglial control of these physiological processes can contribute to neurodevelopmental disorders and neurodegenerative diseases [18].

Neurons and microglia reciprocally modulate their biological status by taking part in several signaling pathways, such as purinergic and adenosine signaling by P2X ionotropic (P2X4 and P2X7) and P2Y metabotropic (P2Y6, P2Y12 and P2Y13) purinergic receptors and A2AR and A3R, Toll-like receptors, widely described in microglia (including TLR-4 and TLR-6), and complement system through C1q, C3 and C5 receptors (CR3 and CR5) highly expressed by microglia [11]. Most notably, C1q and the downstream complement component C3 are critical for the proper elimination of CNS synapses and for the stimulation of the phagocytosis [17]. Moreover, both neurons and microglia can release several factors that enhance cell-to-cell communication. These molecules include neuropeptides, neurotrophins, neurotransmitters and both anti- and pro-inflammatory cytokines and chemokines (Figure 1), involved in several signaling pathways [18].

### 2.1. Cytokines

Cytokines include a large group of small polypeptides that could promptly increase in response to alteration of the physiological state and play a key role in tissue remodeling in pathologic states [19]. These factors and their receptors also play an important role in neuron–microglia crosstalk, being regulators of apoptosis and synaptogenesis. Among them, there are tumor necrosis factor α (TNFα), interferons (IFN), interleukins (ILs), transforming growth factor β (TGFβ), neurotrophic factors (NGF and BDNF) and neurotrophins (NT-3 and -4), colony-stimulating factors (such as M-CSF) and many others.

IL-4, -10, -13 and TGFβ have anti-inflammatory, immunosuppressive and neuroprotective roles. They can be involved in the downregulation of microglial production, as well as blocking the release of pro-inflammatory cytokines, such as ILs and TNF [20,21]. A relevant role has been identified for pro-inflammatory cytokines, including IL-1, IL-6, TNF-α and IFN-γ, produced by microglial cells and involved in neuronal injury and degeneration [19]. Over the last years, neurotrophic factors, such as Nerve Growth Factor (NGF) and Brain-Derived Neurotrophic Factor (BDNF), have emerged as promising candidates for neuron–microglia signaling. Neurotrophins NT-3 and NT-4 have been demonstrated to have neuro-supportive functions, sustaining microglial proliferation and the increase of their phagocytotic activity [21]. They also take part in proper neuronal synaptic plasticity and survival by regulating neurotransmitters release and dendritic growth [18]. Colony-stimulating factors, such as MCSF are considered key factors in glial activation. They control the main microglial properties and biological functions [21], including microglial proliferation, phagocytic ability and the production of reactive oxygen intermediates [22]. In addition to its anti-inflammatory role, TGFβ was shown to reduce Aβ plaques in an animal model of AD by promoting microglial phagocytotic activity [23]. On the other hand, high concentrations of IL-1β, IL-18, IFN and TNFα impair synaptic plasticity with detrimental effects on neurons demonstrating once again the complex balance between neuroprotective and damaging microglial released factors in the microenvironment [11]. Regarding neuroprotection, it has been shown that Il-34 produced by neurons during an inflammatory process contributes to the activation of microglia for the elimination of toxic substances and for the production of antioxidant enzymes [24]. Elevated levels of IFNγ and TNFα have been observed in the injured or ischemic brain or as a consequence of bacterial and viral infections, as well as in case of pathologies, e.g., multiple sclerosis. In particular, increased TNFα levels have been demonstrated to enhance the inflammatory processes [21], as confirmed by its presence in AD brain tissues, together with IL-1 and IL-6. The pro-inflammatory processes mediated by the abovementioned factors strongly contributed to the progressive loss of neuronal structures [25,26].

### 2.2. Chemokines

The superfamily of chemokines consists of a considerable number of proteins with multiple functions in the CNS. They are involved in adult neurogenesis, cell proliferation, synaptic transmission, plasticity and spatial memory. Furthermore, chemokines and their receptors play an important role in controlling microglial activity especially in regulating the inflammatory responses and the development and homeostasis of immune cells [8]. The SDF-1 (also known as CXCL12) and CXCR4 system is prominent for CNS development and for microglial–CNS cells interaction [21]. Additionally, SDF-1-CXCR4 and CCL11 and CCL2 are all involved in promoting microglial migration [27,28]. In order to maintain CNS homeostasis, CX3CL1–CX3CR1 is the most relevant cell-specific ligand–receptor axis, thanks to the unique cell-type-specific localization of its components; in fact, the chemokine fractalkine (CX3CL1), is expressed on neuronal cells while its receptor, CX3CR1, is predominantly expressed on microglial cells.

#### Fractalkine (CX3CL1)

One of the most important pathways involved in microglia–neurons communication is the abovementioned CX3CL1/CX3CR1 axis. The chemokine CX3CL1, also known as fractalkine, is mainly expressed in CNS by neurons and acts through its microglial receptor (CX3CR1) on the activation of PI3K, AKT and NF-κB pathways [29]. Fractalkine exists in both membrane-bound and soluble forms and plays a role in promoting neurogenesis and regulating the number of neurons during normal physiological CNS development through the induction of the microglial release of trophic factors [30]. However, microglial cells may themselves produce fractalkine [31], thus providing an autocrine signaling. The anti-inflammatory properties of CX3CL1 are exerted through the down regulation of the production of inflammatory mediators (iNOS, IL-1β, TNF-α and IL-6) [32] and the suppression of cytokine-induced neuronal cell death. In addition, abundant CX3CL1 has been observed in uninjured adult hippocampi suggesting a functional role for the CX3CL1–CX3CR1 axis in the maturation and elimination of synapses, in the regulation of synaptic transmission and plasticity and consequently in the learning functions and memory formation [30]. Rogers and collaborators have shown several cognitive deficits due to a lack or reduction of CX3CR1 in an in vivo mice model. The authors demonstrated deficits in motor learning in CX3CR1^−/−^ and CX3CR1^+/−^ mice by standard rotarod training techniques. On the other hand, deficits in associative learning and memory in the same mice compared to wild type condition were found by standard fear-conditioning tests, suggesting hippocampal-specific deficits in cognition [33]. Moreover, CX3CR1^−/−^ and CX3CR1^+/−^ mice showed a reduction in LTP in the CA1 striatum radiatum due to more activated microglia and higher levels of inflammatory-released factors as IL-1β in the hippocampus and TNFα in the cerebellum. Treatment of CX3CR1^−/−^ mice with IL-1 receptor antagonist reversed the deficits in learning and memory supporting the crucial role of CX3CL1/CX3CR1 in the homeostasis of synaptic transmission in the hippocampus [33]. Furthermore, several studies suggest that lack or alteration of CX3CR1/CX3CL1 signaling causes a decrease in microglia–neuron communication that leads to the loss of anti-inflammatory function of microglia [11]. This is associated to an increased production of pro-inflammatory molecules with an increased risk of neurodegenerative diseases and neurodevelopmental disorders [8]. In the genetic ALS model (G93ASOD1) of spinal cord motoneuronal death, CX3CR1 deficiency was accompanied by higher levels of microglia activation and by the parallel increased neuronal death, compared to wild-type mice, where the neurotoxic activity of microglial cells was prevented by the CX3CL1–CX3CR1 axis [34].

### 2.3. Immunoglobulins Superfamily (IgSF)

Among the various types of molecules involved in cell–cell interactions in the nervous system, the immunoglobulin superfamily (IgSF) must be mentioned. IgSF is a large versatile family of cell surface and soluble proteins involved in different types of interactions and in the structural composition of proteins. In addition, IgSF domains take part in the morphogenesis, maintenance and regeneration of neurons, as well as in the synaptogenesis and myelination [35]. The best characterized molecules belonging to this family in the CNS are TREM2 and CD200 expressed on the membrane surface of microglia and neurons, respectively.

#### 2.3.1. TREM2

TREM2 (Triggering Receptor Expressed on Myeloid cells-2) is an innate immune receptor of the CNS, member of the immunoglobulins superfamily (IgSF) expressed on the membrane of microglia, regulating several functions. Worthy of notice, TREM2 expression needs the involvement of DAP12 (DNAXactivating protein of 12 kDa) for the development of functional neuronal synapses [10], which is also involved in maintaining the CNS immune homeostasis by blocking microglial inflammatory activity [20]. The production of microglial proinflammatory mediators TNF-α and iNOS (inducible nitric oxide synthase) was reduced by TREM2 activation with consequent protective effects on neuronal damage. Furthermore, TREM2 activation increased microglial phagocytic activity promoting the removal of apoptotic neurons, damaged cells, organic matrix components and macromolecules [12]. In murine models of multiple sclerosis and autoimmune encephalomyelitis, an intensified phagocytic activity was related to an increased expression of TREM2 on microglia [36]. At the same time, the reduction or the lack of TREM2 in BV2 microglia cell and in in vivo model, reduced phagocytosis in oxygen–glucose-deprived neurons in vitro and likewise in an experimental stroke model [37]. Therefore, mutations of TREM2 are associated with increased risk of neurodegenerative diseases, including neurodevelopmental disorders, such as autism. Autistic patients have shown reduced levels of TREM2 [38]. In addition, an in vivo study has confirmed sociability defects and altered brain connectivity in a mouse model lacking this protein [39]. A key role of TREM in AD have been confirmed too. Overwhelming evidence suggests that TREM2 expression is promoted in the brain of AD patient in parallel to Aβ accumulation, increasing the phagocytosis of amyloid plaques by activated microglia [12]. In addition, an improvement in the Aβ deposition, neuroinflammation, neuronal and synaptic loss has been observed in brains of APP/PS1 mice due to overexpression of TREM2 [36].

#### 2.3.2. CD200

Besides being a member of the superfamily of immunoglobulins, CD200 (cluster of differentiation-200) belongs to the clusters of differentiation (CD), a class of multiple cell surface proteins that includes cell markers used in immunophenotyping. These proteins are involved in several physiological functions, including cell signaling and adhesion, proliferation or migration, apoptosis, neurodevelopment and synaptic plasticity. In the central nervous system, one of the main functions of CD factors is the regulation of microglial activity. Indeed, specific CD proteins are considered markers of specific microglial phenotypes reflecting the activated state of these cells. For example, M1 microglia are characterized by the expression of CD14, CD16, CD32, CD40, CD45, CD68, CD74 and CD86, while M2 microglia are mainly associated with CD23, CD33, CD36, CD64, CD68, CD80, CD86, CD163, CD200R, CD204, CD206 and CD209 [40]. The interaction between CD200—mainly expressed by neurons—and its receptor (CD200R) detected in microglia, is associated with the regulation of phagocytic activity of the latter.

The CD200–CD200R axis keeps the microglial cells in a non-activated state [12,32]. Viral-mediated re-expression of CD200 in APP mice reduced neuroinflammation restoring microglial phagocytic function and enhancing synaptic plasticity [11]. In addition, this signaling axis is involved in the production of the glial cell-derived neurotrophic factor (GDNF), a survival factor for dopaminergic neurons [8]. Furthermore, the CD200–CD200R axis was demonstrated to be regulated by a cytokines feedback mechanism [12,32]. Cytokines released by proinflammatory stimuli downregulate CD200 expression while certain cytokines could increase or preserve its expression, such as IL-4 [41]. In an in vivo model of AD, IL-4 treatment restored CD200 expression with the maintenance of phagocytic response and an increase of Aβ clearance [42]. A deficit of the CD200–CD200R complex was then associated with the activation of microglia responsible for the increased levels of IL-1β, IL-6 and TNF-α, in both AD patients and mouse models [11].

### 2.4. Role of Extracellular Vesicles in Neuron–Microglia Crosstalk

Neuron–microglia communication is a complex mechanism finely regulated by the above-described factors expressed and or released by cells according to perceived stimuli. In this intricate system, released factors are growing in importance as information carriers not only under physiological condition, in which they are necessary to maintain the brain homeostasis, but also in case of pathology. The family of released factors includes soluble molecules directly released in the extracellular space, as well as mediators released through vesicles. Extracellular vesicles (EVs) are taking hold in recent years both as vehicle to spread inflammatory mediators especially in the neurodegenerative diseases characterized by the “non-cell autonomous” mechanism and as a promising therapeutic way to counteract the neuronal damage.

Living cells constantly release vesicles that differ in size, content and biogenesis according to their different associated functions. According to their size, it is possible to distinguish three different categories of EVs: exosomes (30–150 nm), the smallest vesicles that follow the endosomal path; microvesicles (also known as ectosomes and microparticles) (0.1–1 μm) produced by the cell membrane, which incorporate cargoes to be transported, and apoptotic bodies (0.8–5 μm), characteristic of dying cells [43]. For a long time, vesicles have been considered only as cellular debris. Recent studies have assigned a leading role to exosomes as a novel mode of intercellular communication, which may be performed in many physiological and pathological processes including immune response, signal transduction and neuroinflammation [44,45]. Donor cells (microglia, neurons, astrocytes and oligodendrocytes) release EVs not only for intercellular communication, but also for the removal of toxic molecules from the cytoplasm. The content of cell-derived EVs includes proteins, lipids and nucleic acids and is briefly described below. Most vesicles contain pro-inflammatory cytokines, such as IL-1β, interferon-γ, tumor necrosis factor (TNF) and chemokines, as neuronal CCL21 that is transported into neuronal processes reaching presynaptic terminal [46,47].

#### 2.4.1. Proteins

Proteomic studies have revealed that exosomes are enriched of several proteins mainly involved in vesicle structure, biogenesis and trafficking. Among these proteins, there are tetraspanins (CD9, CD63, CD81 and CD82,) involved in cell penetration, invasion and fusion events; heat shock proteins (Hsp70 and Hsp90), implicated in the stress response and in antigen binding and presentation; the endosomal sorting complexes required for the transport complex and exosome release (Alix and TSG101). Furthermore, proteins responsible for membrane transport and fusion (as annexins and Rab), cytoskeleton proteins (actin, tubulin, etc.), metabolic enzymes and ribosomal proteins are part of the protein components found in exosomes [44,48]. The study carried out by Glebov demonstrated both in vitro and ex vivo that stimulators of serotonin (5-HT) receptors increased the release of an insulin-degrading enzyme associated with microglial exosome shedding involved in degradation of the neurotoxic peptide amyloid-β [49]. In the CNS, EVs are also indicated as potential carriers of misfolded proteins involved in neurodegenerative disorders, such as Tau and amyloid β in AD, α-synuclein in PD, TAR DNA-binding protein 43 (TDP43) and copper zinc superoxide dismutase 1 (SOD1) in ALS, suggesting their possible role as potential biomarkers for different neurodegenerative diseases [50,51].

#### 2.4.2. Lipids

The activity, stability and structural rigidity of exosomes are guaranteed also by their lipid components. Exosomes are enriched in membrane lipids, as sphingomyelin, phosphatidylcholine, phosphatidylethanolamine, phosphatidylserine, ganglioside GM3 and phosphatidylinositol, prostaglandins, cholesterol, arachidonic acid and other fatty acids, but also in some functional lipolytic enzymes [44]. In addition to their functional role in maintaining EVs structure and stability, lipids also facilitate the fusion and fission of the EVs, as demonstrated by their involvement in EVs biogenesis through the modulation of intracellular fusion and budding processes [48]. It has been shown that microvesicles deriving from microglia induce an amplification of the frequency of postsynaptic excitatory current independently of the release of cytokines but by inducing the metabolism of sphingolipids in neurons [52]. Gabrielli and collaborators demonstrated that EVs secreted by microglial cell contain endocannabinoids that can stimulate type-1 cannabinoid receptors (CB1) and inhibit presynaptic transmission of GABAergic neurons [53].

#### 2.4.3. Nucleic Acids

As a subtype of EVs, it is worth mentioning exosomes for their content of different nucleic acids. MicroRNAs (miRNAs) are the most abundant derived exosomal RNA species, but also ribosomal RNA (rRNAs), long non-coding RNA (lncRNAs), transfer RNA (tRNAs), small nuclear RNA (smRNAs), viral RNA and single and double-stranded DNA have been found in exosomes [54]. All these nucleic acids could have a great impact in different biological processes including oxidative stress, inflammation, apoptosis, blood–brain barrier protection, angiogenesis and neurogenesis [44]. MicroRNAs (miRNAs) are small (18–24 bp) noncoding single-stranded RNAs involved in post-transcriptional and post-translational regulation by binding to specific sites within the 3′ untranslated region (UTR) [55]. Three categories of extracellular miRNAs can be distinguished: (i) circulating detected in the extracellular environment, including in biological fluids (such as urine, blood, tears and cerebrospinal fluid); (ii) mainly transported by EVs; and (iii) those associated to other carriers (HDL (high-density lipoprotein) and Ago protein).

Several findings, including an in vivo study, suggest a specific involvement of miRNAs in physiological cell-to-cell communication. The content of exosomes both released and taken up by dendritic cells showed the presence of miR-155 and miR-146a, two critical inflammation-related miRNAs able to modulate microglia phenotype [56]. Likewise, another recent study indicated a dialog between microglia and neurons mediated by miR-1860, miR-7718, miR-2284y6 and miR-146a delivered by microglial EVs to neurons. Even though these miRNAs were poorly expressed, they played an important role by modulating essential biological processes [57].

Prada and collaborators highlighted the release of miR-146a-5p through EVs from microglial cells to neurons: They showed that vesicular miR-146a-5p could control the expression of presynaptic synaptotagmin1 (Syt1) and postsynaptic neuroligin1 (Nlg1), two proteins with a central role in dendritic spine formation and synaptic stability [58].

Another notable example of miRNAs involvement in the regulation of essential functions in the CNS was reported by Morton and colleagues. They observed an abundant presence of miR-9, Let-7, miR-26 and miR-181 in NSCs (Neuronal Stem Cells) EVs. Their uptake by microglia has been proven to be responsible for transcriptional modification of genes involved in the regulation of microglia morphology, immune response, chemotaxis and cytokine production [59].

Recently, Pinto and collaborators performed an in vitro study on the motoneuronal cell line NSC-34 that over-expresses SOD1 with mutations linked to ALS. They found that exosome shedding from these miR-124-containing cells could play a crucial role in the microglial polarization from pro-inflammatory phenotype M1 to the anti-inflammatory phenotype M2 [60]. Song and colleagues investigated the content of miR-124-rich exosomes released by microglia both in vitro and in vivo. Interestingly, these exosomes were taken up by neurons and mir-124 targeted and reduced the expression of ROCK and USP14 promoting neuroprotection in brain damaged by ischemic stroke [61]. Recent research reveals that miR-124 enriched microglial exosomes promoted neuronal regeneration in hippocampus after TBI (Traumatic Brain Injury) by inhibiting the TLR4 signaling pathway and producing M2 microglial polarization. Dysregulation of miRNAs has been shown to contribute to many types of human diseases, including neuronal disorders [62]. MiRNAs circulating in extracellular space are stable and detectable through various techniques, which makes them potential diagnostic biomarkers for several of these diseases [55]. For example, miR-21 detected in the cerebrospinal fluid EVs has been proved as a biomarker for glioblastoma development [63].

Zhang and collaborators discussed the indirect effect of miR-181c on neurons. MiR-181c seems to promote the overexpression of TNF-α in microglial cells resulting in a microglia-mediated neuronal injury [64]. The gain and/or loss of function of microglial cell in neurodegenerative diseases is a well-known mechanism capable of spreading inflammatory markers to neighbor cells, as well as altering the microenvironment homeostasis. It is turning out that the “non-cell autonomous” mechanism of neurodegeneration, mostly described in ALS, might be caused by inflammatory mediators release not only directly in solution, but mainly through vesicles from activated microglia. It has been demonstrated that overexpressing human SOD1 with ALS-linked mutations in microglia promoted the increase in the pro-inflammatory markers expression and cytokine release compatible with activated cells and the following release into exosomes of pro-inflammatory miRNAs [65]. When the N9 microglial murine cell line was made to over-express the wild-type human SOD1, it showed a high expression of miRNA-146a. Still, this miRNA was detected only in exosomes when N9 microglia was made to overexpress human SOD1 with ALS-linked mutations. MiRNA-155 was also found in exosomes, thus further contributing to the dissemination of inflammatory mediators. The role of miRNAs in microglial exosomes on regulating neurodegeneration was also demonstrated in repetitive mild TBI, important risk factor for long-term neurodegenerative disorders, such as AD. In vivo, hyperphosphorylation of Tau was reduced via injection of EVs with overexpressed miRNA-711 secreted by BV-2 human microglia cells [66]. This has been suggested to affect the neuro-immunological interactions between macrophages and sensory neurons [67]. Similarly, the uptake of exosomes enriched in miRNAs released by human neuroblastoma cells (SH-SY5Y) overexpressing the *APP* gene with AD-linked mutations resulted in a comparable increase of miR-155, miR-146a and miR-124 in the human microglial cell line (HMC3), as well as in the upregulation of pro-inflammatory markers, including IL-10 and Arginase 1 expression [68].

Given the crucial role of miRNAs in physiological and pathological molecular pathways as in cell-to-cell communication, an increasing number of studies have revealed their possible use as important therapeutic targets. For example, in vitro, the levels of APP and β-amyloid in damaged neurons were restored through the treatment with exosomes enriched with miR-124-3p, whose expression in microglia was shown to be significantly altered in the acute, sub-acute and chronic phases after rmTBI [69]. Finally, memory ability and behavior were significantly improved in APP/PS1 double transgenic mouse model of AD through the injection of miRNA-22 mimic that also attenuated the activation of NLRP3 inflammasome and the expression of inflammatory factors in mouse hippocampus, as well as the expression of Gasdermin-D, a critical mediator of innate immune defense [70].

## 3. Epigenetic Mechanisms Involved in Microglia Activation

Generally described as being responsible for the environmental surveillance, microglia shift their resting state into an activated one, as a result of different environmental stimuli. At the first instance, the main detected activated phenotype is the M2 anti-inflammatory one, aimed to protect the surrounding environment form damage. Neurodegenerative diseases are characterized by a chronic inflammatory condition promoted by the M1 pro-inflammatory phenotype of microglia that enhance the neuronal damage in a cause-and-effect mechanism not yet clearly explained. The coordinated modification of microglial phenotypes is finely regulated by modulation of genes expression, resulting in changes in chromatin structure and composition, by action of epigenetic modulators. Therefore, histone post-translational modifications (i.e., methylation, acetylation and phosphorylation), DNA methylation or gene expression regulation by non-coding RNAs are crucial to control microglia plasticity and polarization into specific phenotypes, as well as their activation states in health and disease [71].

### 3.1. DNA Methylation

DNA methylation is a modification occurring on the cytosine residues of CpGs dinucleotides through the addition of a methyl group, generally associated with the inhibition of transcription. Even if not fully understood, cytosine methylation (5mC), together with DNA hydroxymethylation, turns out to be essential in regulating gene expression in the aging brain [72,73,74,75]. However, in contrast to the well-known role of histone acetylation, little is known about gene expression regulation by DNA methylation in microglia development and function. Cho and colleagues (2015) reported that the expression of the inflammatory cytokine interleukin-1 (IL1β) in the CNS was strictly dependent on DNA methylation in aging microglia, with the consequent upregulation of IL1β production following hypomethylation of specific CpGs sites on IL1β promoter in two different models of aging [76]. Subsequently, Matt and coworkers (2016) confirmed the role of DNA methylation in microglia activation by treating BV-2 and primary microglia cells with 5-azacytidine, a DNA methylation inhibitor, which increased *IL1β* gene expression [77]. A significant decrease of 5mC and of the hydroxymethylation level of 5mC was detected in both glia and neurons, in the hippocampus of AD patients [78,79,80]. In contrast, Bradley and colleagues demonstrated an increased global level of both methylated and oxidized 5mC in brain areas associated with memory and cognitive function [73]. Additionally, in other well-known pathologies, like HD [81], dementia with Lewy bodies, PD disease [82] and ALS [67,83], it has been demonstrated that the DNA methylation pattern is altered, with a strong impact on microglial phenotype and function.

Furthermore, it has been showed that the inhibition of methyltransferases in BV-2 microglia cells, increased the production of amyloid-β via the promotion of demethylation in the promoter region of presenilin 1 (PSEN1) and BACE1 expression as well [84]. Two specific CpG sites in positions +298 and +351 in the 5′UTR of the *BACE1* gene have been identified in BV-2 cells as targets for the epigenetic modulation of its expression in microglia [85]. In fact, BACE1 inhibition is one of the most important therapeutic approaches of AD in order to reduce the generation of the neurotoxic β amyloid protein [86] and to promote the immunomodulation of microglia with neuroprotective effects [87]. In eight-month-old male 5XFAD mice treated with a histone methyltransferase inhibitor, an in vivo model of AD, the reduction of DNA methylation and the increase of hydroxymethylation turned into the reduction of β-amyloid plaques and into the rescue of cognition impairment and locomotor activity [88]. Moreover, gene expression of neuroinflammation markers, such as Il-6 and TNF-α, was decreased in treated mice, demonstrating the neuroprotective and anti-inflammatory effect of DNA hypomethylation in neurodegenerative diseases.

### 3.2. Histones Post-Translational Modifications

Chromatin accessibility by transcriptional complexes is closely related to post-translational modifications occurring on the histone amino (N)-terminal tails, which define chromatin as heterochromatin (condensed state) or euchromatin (non-condensed or open state). The main histone modifications include methylation, acetylation or phosphorylation. Histone methylation is associated to either transcription activation or repression, depending on the amino acid in which the modification occurs. Histone methyltransferases (HMTs) promote mono-, di- or tri-methylation on histone residues, whereas histone demethylases (HDMs) act to remove methyl groups from target proteins. Conversely, histone acetylation is regulated by histone acetyl transferases (HATs), which acetylate the lysine residues on histones tails or core promoting gene transcription. Histone deacetylases (HDACs) remove the acetyl groups from lysine residues, thus leading to the silencing of gene expression. Focusing on the role of histones acetylation and deacetylation in regulating microglia dynamic processes, the interplay of HATs and HDACs is crucial for the proper CNS homeostasis. In the context of inflammatory response, HDAC1 and HDAC2 showed functional redundancy and the upregulation of HDAC2 expression turned out to compensate for HDAC1 deficiency [89]. HDAC1 and HDAC2 are crucial also for microglia development. Datta and colleagues showed that the contemporary *HDAC1* and *HDAC2* gene depletion in vivo leads to different alterations in microglia during development or neurodegeneration. In fact, in adult microglia HDAC1-2 deletion had no effects on cell number or morphology during homeostasis, whereas in an AD mouse model the deletion positively affected microglial phagocytosis of amyloid plaques [90]. However, after HDAC1 and HDAC2 downregulation, genome-wide profiling for histone H3K9 and H3K27 acetylation revealed only a slight increase in global acetylation levels at the promoters of genes regulating cell cycle and microglia activation (e.g., Cdkn2c, Ifnar2 and Sema6d) [90], thus indicating that the role of HDAC1-2 in microglia need to be investigated more. Since transcriptional dysregulation is an important hallmark of neurodegenerative diseases, HDACs inhibition has been extensively investigated as a therapeutic strategy to promote neuroprotection and inflammatory response [91,92,93,94,95]. Most studies focused on the use of pan-HDAC inhibitors, which do not have a selective mechanism of action. In particular, Trichostatin-A (TSA), one of the most known HDACs inhibitors, strongly inhibited the nitric-oxide production and decreased the mRNA and protein levels of proinflammatory cytokines, such as TNF-α, IL-6 and IL-1β in RAW264.7 cells and bone-marrow-derived macrophages. The pretreatment with TSA increased the level of the anti-inflammatory cytokine IL-10, instead [96]. Comparably, another known pan-HDAC inhibitors, vorinostat, strongly suppressed the LPS-induced cytokine expression and release in primary microglia and in microglia acutely isolated from LPS-treated mice [97]. The neuroprotective role of HDACs modulation had been already proven in the rotenone rat model after chronic administration of valproic acid (VPA), a pan-HDAC inhibitor, paving the way for epigenetic modulation of neurodegenerative disease [94].

However, it turns out that the inhibition of different HDAC isoforms might have different therapeutic potentials. A recent study on BV-2 cells and in vivo strongly demonstrated the effect of a specific HDAC-8 inhibitor, WK2-16, on the neuroinflammation promoted by LPS [98]. WK2-16 significantly suppressed the LPS-induced expression of COX-2 and iNOS and TNF-α production in BV-2 cells via Akt and STAT-1/-3 inhibition already demonstrated to attenuate the inflammatory response. The beneficial function of HDAC-6 inhibition against neurodegenerative diseases was demonstrated in AD trough the inhibition of Tau phosphorylation [99] in SOD1G93A mouse model of ALS [100,101] and in iPSC-derived motor neurons from ALS patients with FUS mutations [102]. Recent findings demonstrated that HDAC-6 inhibition could increase the sigma-1 receptor (Sig-1R) expression in primary microglia [103]. Its allosteric modulation attenuated the expression of pro-inflammatory markers, such as TNF-α, IL-1β and iNOS; the release of NO; and the production of ROS in LPS-stimulated BV-2 cells [104]. The specific inhibition of HDAC-2 also reduced the levels of inflammatory cytokines TNF-α and IL-1β in LPS-activated BV-2 microglial cells and LPS-treated mice through attenuating the TLR4/NF-κB signaling pathway, confirming the promising role of HDACs inhibition in the attenuation of microglia inflammation [105]. The promising effects of HDAC inhibition on various in vitro and in vivo model of classically activated microglia (M1-phenotype) support the potentially neuroprotection mechanisms through epigenetic immunomodulation of microglia [106,107,108,109].

### 3.3. MicroRNAs (miRNAs)

Microglial “resting” state, as well as the M2/M1 activation states, can be characterized by the expression of different microRNAs (miRNAs), which are involved in many cellular pathways, including proliferation, differentiation, apoptosis and cellular communication [110]. In the cells, miRNAs mainly regulate the silencing of gene expression by driving the degradation of target mRNA and triggering translational repression [111]. MiRNAs expression in cells can be coordinated by regulation mechanism at both transcriptional and post-transcriptional levels and also through the action of endogenous and exogenous molecules [112]. Several miRNAs are targeted to be exported and, after the recognition, they are taken up by neighboring or distant receiving cells and utilized to modulate the gene expression [113]. Accumulated evidences showed that miRNAs exosomal packing process does not happen randomly, but is based on four possible mechanisms involving the neural sphingomyelinase 2 (nSMase2), the sumoylation of heterogeneous nuclear ribonucleoproteins (hnRNPs), the 3′-end of the miRNA sequence-dependent pathway or the miRNA induced silencing complex (miRISC)-related pathway [114]. As already described, miRNAs can be delivered in EVs and, depending on the donor cell status, can act on physiological mechanisms or amplify a pathological condition of recipient cells [113]. In the last years, their role in the modulation of macrophage polarization and inflammation has been deeply investigated, as well as their use as biomarker for neurodegenerative diseases.

Using primary microglia exposed to LPS (M1-phenotype) or IL-4 (M2-phenotype), Freilich and colleagues identified a different miRNA expression pattern according to microglia polarization [115]. The microarray expression profiling and the bioinformatics analysis of miRNAs identified the miRNA-155 as the most significantly upregulated in the pro-inflammatory state confirming a previous study [116]. In addition, upregulation of miRNA-145 and downregulation of miR-711 and mir-124 was strongly associated with the alternative activated phenotype of microglia (M2) promoted by IL-4 [115].

Despite the fact that several miRNAs have been associated with different microglia polarization profiles, miRNA-146a was demonstrated to be an important regulator of the inflammatory response [117,118,119]. Peritoneal macrophages overexpressing miR-146a showed a reduced expression of M1-associated proteins and an increased expression of M2-ones after LPS treatment [119]. Furthermore, LPS-stimulated miRNA-146a knock-out (KO) microglia showed a significant increase of M1 phenotype markers and a reduced migration and phagocytic activity in KO compared to wild-type (WT) microglia [117] confirming the anti-inflammatory profile of miRNA-146a. In addition, the effect of Resveratrol (RSV) in reverting the LPS-induced phenotype from M1 to M2 subtypes [120] was demonstrated to be dependent on miRNA-146a-5p since the reduction of TNF-α, IL-1β and IL-6 level by RSV was reversed by miR-146a-5p silencing [121]. Furthermore, the upregulation of miRNA-146a and miRNA-155 in BV-2 cells was also inhibited by cannabinoids via the NF-κB signaling, followed by the up-regulation of miRNA-34a and the modulation of Notch signaling pathway [122]. The inactivation of the LPS-dependent NF-κB signaling, as well as the pro-inflammatory mediators iNOS, TNF-a and IL-1b in BV-2 cells, was also promoted by curcumin, which increased the level of miR-199b-5p and decreased the inhibitor of NF-κB kinase subunit beta IKKβ expression [123]. Among the canonical LPS inflammatory stimulus acting on microglia, P2X7 receptor activation trough ATP stimulation has been shown to increase miRNA-125b in microglia [124] and consequently the inflammatory mediator NF-κB [125,126], which was then decreased by miRNA-125 inhibition in microglia overexpressing the human SOD1 with ALS-linked mutations [124,127].

## 4. Neuronal-Mediated Epigenetic Reprogramming of Microglia in CNS Health and Disease

As reported above, to survey the CNS microenvironment and maintain proper homeostasis, microglia actively interact with neurons and astrocytes, which release a wide variety of soluble factors that could affect microglia maturation, development and phenotype [128]. Ayata and colleagues have reported that microglia clearance activity is regionally regulated, depending on the amount of dying neurons and non-functional synapses in cerebellar, striatal and cortical brain. Exposure to apoptotic cells induces pro-phagocytic genes and transcription factors expression, facilitating engulfment and removal of cells and cellular debris with parallel downregulation of genes involved in homeostatic surveillance. As recently demonstrated, following neuronal death, *Kdm6a/b* gene in microglia encodes the histone demethylase KDM6A/B, which removes H3K27me3 previously induced by the Polycomb repressive complex 2 (PRC2) to the promoters of clearance genes [129,130,131]. Neuron–microglia crosstalk is then crucial to maintain the CNS physiological condition and to prevent the onset and the progression of neurodegenerative diseases; microglia support neuronal maturation and synaptic networks, whereas neurons provide specific factors that induce epigenetic changes in microglial chromatin, regulating their phenotypes and functions. To allow lineage-specific differentiation, as well as the survival and renewal of microglia in brain tissues, neurons and astrocytes, produces specific factors, such as the macrophage colony-stimulating factor-1 (CSF-1) and interleukin (IL)-34 [132], which induce expression of microglial-specific genes, i.e., Mafb, Mef2c, Sall1 and Spi1 [133]. SPI1 (human) or Spi1 (murine) encode for PU.1, an ETS-domain transcription factor, which binds PU-box (purine-rich sequences) and activates microglial-specific genes expression regulating several microglial functions [134,135]. ATAC-seq analysis on microglia at different temporal stages showed the PU.1 promoter highly upregulated by histone H4 acetylation, further confirmed by histone deacetylate (HDAC) inhibition which led to the suppression of UP.1 transcription, followed by the increase in H4 acetylation levels and the disruption of locus and RNA polymerase II interaction [136,137]. However, many other factors as interferon response factor (IRF)-8, V-Maf musculoaponeurotic fibrosarcoma oncogene homolog B (MAFB) and peroxisome proliferator-activated receptor (PPAR)γ, transcriptionally regulate microglia identity in healthy CNS [138,139]. Therefore, neuronal-induced expression of several microglial-specific transcription factors (PU.1, CEBPα, IRF8 and SALL1), leads to chromatin modification and generation of enhancers for gene expression, such as histone H3 K4monomethylated (H3K4me1) and histone H3 K9lysine acetylation (H3K9ac). On the other hand, inflammatory stimuli, e.g., IL-6 and tumor necrosis factor (TNF), induce NFκB, NF-AT and STAT1/3 expression, and increase histone H3 k27acetylation (H3K27Ac) [139]. However, like the acquisition of a mature microglial-specific phenotype, microglia activation and polarization required epigenetic changes that are associated with both M1 and M2 microglia phenotype [140,141]. CNS-derived IL-4 production via neurons, and possibly astrocytes, contributed to M2-like markers and M2-associated transcription factors expression, e.g., PPARγ in mature microglia [142] or the macrophage inflammation protein Ym1 [132]. More specifically, IL-4 induces H4R3 methylation and regulate acquisition of M2 microglia-phenotype by inducing PPARγ expression in mouse peritoneal macrophages (PMs) [143], whereas histone H3 K4methylation (H3K4me), induces M2 polarization in human macrophages following M-CSF and IL-4 stimulation [144]. Additionally, IL-4 activated mouse microglia have been reported to upregulate H3K27 demethylase JMJD3, leading to H3K27 demethylation essential for IRF4 and Arg1 overexpression [145]. Therefore, following neuronal stimuli, multiple transcription factors in microglia induce epigenetic changes, leading to remodeling of chromatin and formation of specific microglial phenotype, with possible consequences on neuronal activity/maturation and synaptic networks.

## 5. Studying Microglia–Neuron Crosstalk with Advanced Microscopy Techniques

In studying complex biological systems, such as the neuroimmune system, spatial and temporal resolution are important requirements to get reliable information about changes in crosstalk and cell morphology in pathological and physiological conditions. Microscopy evolved, aiming to fulfill both needs, and developing new methodologies in which both dynamicity and morphology may be described as precisely as possible [146].

Intravital microscopy (IVM) allows the achievement of temporal resolution in microglia imaging in brain tissue. In animal model, non-invasive in vivo high-resolution techniques are the best choice to observe dynamic processes involving microglia. IVM allows the analysis of physiological and pathological processes at microscopic resolution in living animals, also for prolonged time (weeks). These techniques applied to brain characterization are extensively described in the literature [147,148,149,150,151]. In such approaches, a craniotomy of the animal model is required to get direct access to the brain tissue. One of the most common approaches is the “open skull” technique, in which a circular portion of the skull is removed, and a cover glass is applied on the area of analysis [150]. IVM can be combined with different imaging strategies that are intended to investigate in vivo phenomena.

Confocal microscopy allows the characterization of the morphology of microglia in three dimensions (resolution ~0.8 μm in *x*–*y*, ∼0.3 μm in *z*) by acquiring Z-stacks of the samples. This technique provided information about the interaction of microglia and other cell types in pathological and physiological conditions elucidating 3D structures [152,153,154]. Although confocal microscopy has been applied for in vivo imaging, it is affected by limited resolution at deeper depths due to scattering and emission from surrounding out-of-focus sample [155].

The technique of choice of in vivo imaging of neuroimmune system is multi-photon microscopy (MPM), which has been significant for studying microglia functional dynamics in brain tissue, and elucidating interactions with neurons, blood vessels, astrocytes and immune cells in real-time [156,157]. First observations in intravital microscopy showed that microglia were unexpectedly dynamic. In vivo imaging with MPM allowed the morphological characterization of resting and activated microglia, revealing a ramified morphology even in the resting state with highly mobile protrusions extended towards a damaged area [4,158]. Multi-photon microscopy is based on the application of two or more low-energy near-infrared photons (wavelength above 700 nm) to induce an electronic transition comparable to the absorption of a photon with double energy. The spatial resolution is about 0.45 μm for *x*–*y*, and 0.85 μm for *z*. The application of multiple photons with lower energy reduces the phototoxicity and eventual sample damages in the focus region and permits the achievement of a deeper penetration with a reduced out of focus fluorescence. The potential of such techniques was proved imaging in vivo resting microglia processes making contacts with neuronal synapses over time, depending on the neuronal activity, unveiling a reciprocal regulation [159,160]. Chen and coworkers used two-photon microscopy to discriminate the migration of microglia compared to other tumor-associated macrophages in glioblastoma affected tissues [161]. Many other examples can be found in the literature, in different contexts, from cancer to neurodegenerative diseases [157,160,161,162,163,164,165,166].

Temporal resolution may be achieved also by speeding up the imaging. Light-sheet microscopy has been employed to perform high speed scanning of large brain tissue volumes, with a basic fluorescence setup. A sheet of laser light is employed to get illumination on the sample and detection is obtained traverse to the illumination. This is a non-destructive imaging technique, preventing sectioning artifacts due to cells and slices over-manipulation. Although spatial resolution is generally low (depending on the detection objective), the combination with super-resolution techniques and the development of more sophisticated variants [146,167], brought lateral resolution (*x*–*y*) close to 100 nm and axial resolution (*z*) to about 0.15–0.3 μm with a potential imaging speed of 2.7 × 10^4^ μm^3^·s^−1^, allowing the prevention of photobleaching [146]. With this technique, it is usually possible to visualize an entire adult mouse brain in physiological and pathological conditions, though in vivo applications on whole mouse brain are not yet reported [168,169].

In dealing with spatial resolution, super-resolution microscopy gives a great contribution in studying interactions of microglia with synapses. STED (Stimulated Emission Depletion) microscopy overcomes light-diffraction limited fluorescence microscopy imaging. The samples are point-scanned, using a traditional diffraction-limited focused beam together with a doughnut-like beam to deplete the fluorophores on the outer part of the excitation spot, and thus only the central area of the spot is read, enabling a resolution of about 65 nm in *x*–*y* and 150 nm in *z*. Ormel and coworkers detected postsynaptic material inside and in proximity of microglia in cerebral organoids, using STED microscopy [170]. STED microscopy approaches have been adopted to improve two-photon microscopy to image the morphology of dendritic spines and microglial cells below the surface of acute brain slices [162,171]. Although excitation may often damage samples and although the setup could be complex, STED can provide excellent results even with high-speed imaging [172]. STORM (Stochastic optical reconstruction microscopy) and PALM (Photoactivated Localization Microscopy) are super-resolution optical microscopy techniques based on stochastic switching of the fluorescence of single molecules. These are based on the use of fluorescent probes that can switch between fluorescent and dark states. Only a small but optically detectable fraction of the fluorophores would be detected in every snapshot, allowing the precise determination of their position. The super-resolution image is reconstructed from the spots accumulated over many micrographs. Resolution can reach 30 nm in *x–y* and 50 nm in *z*, though special fluorophores are required and phototoxicity is an issue after multiple imaging cycles. Cserép and coworkers combined two-photon microscopy with high resolution STORM microscopy to understand the effective communication of microglia with neuronal cells and to show the recruitment of microglia towards neurons infected by alpha-herpesvirus [164,173]. Recently, confocal and super-resolution microscopy showed how spreading depolarization induce rapid morphological and positional changes of microglia in ischemia [174]. These are a notable example of how the combination of such techniques may return a great amount of information in studying in vivo brain processes. Among super-resolution techniques, also structured illumination microscopy (SIM) has been applied for such studies. SIM is a fluorescence microscopy technique in which the resolution is enhanced by using the information obtained from frequency space out of the region of interest, using a Fourier transform and collecting superimposed information, afterwards separated through computational analysis [167]. Although nanometric resolution is achieved (90–140 nm), with no special sample preparation, computational procedures may be challenging. SIM has been applied to image mice brain to assess microglia phagocytosis and synapses pruning in injured brain tissue [175,176].

Although not a choice for in vivo studies, electron microscopy (EM) and its derived techniques have been important in microglia characterization. EM can show a considerable number of details in the analyzed sample, from cells to proteins structure even at atomic scale. These ultrastructural techniques may return crucial details at high resolution, revealing the communications between microglia and synaptic elements, helping in the definition of the role of microglia in synaptic pruning, especially if combined with other techniques, such as multiphoton microscopy [166,176].

Correlative light and electron microscopy (CLEM) has brought minimally invasive optical in vivo imaging (usually fluorescence) together with the nanometer resolution of serial section TEM (Transmission Electron Microscopy) or similar EM techniques. A large amount of information about structures and dynamics of the neuroimmune cells can be obtained from the same sample [177,178]. Weinhard and coworkers used CLEM to perform a 3D ultrastructural characterization of organotypic hippocampal cultures, enriching the understanding of the dynamic interaction of postsynaptic spines with microglia [179]. Intravital CLEM pairs the high spatial resolution of electron microscopy with the improved temporal resolution of in vivo microscopy techniques, such as intravital multiphoton microscopy [180]. Intravital two-photon microscopy combined with scanning transmission electron microscopy (STEM), helped in the study of the chronology of the neuroinflammatory events right after the occurrence of an injury, showing the role of resident microglia and amoeboid microglia in brain tissues [181]. Correlation between optical and electronic microscopy images is often challenging, but strategies to improve this aspect are being explored [182].

Great perspective may be expected for techniques such as intravital CLEM, in which in vivo noninvasive imaging with temporal resolution is matched to high spatial resolution electron microscopy. Such combinations respond to the needs previously anticipated for the imaging of complex neuroimmune system and its cellular effectors, such as microglia. It is undoubtedly clear that the study of microglia–neuron crosstalk asks for different observations and, thus, techniques, brought together and integrated to exhaustively depict the entire picture.

## 6. 3D-Cell Culture Systems as Models for The Interaction of Microglia and Neurons

On top of their long-known role as the innate immune cells of the CNS, microglia appear to have additional important roles, such as controlling the number of neuronal precursor cells and modulating synapse formation and elimination [179,183,184,185]. Due to this, it is crucial to have cell models that can recapitulate the physiological 3D state of microglia and the interactions of microglia with neurons and macroglia. It is currently accepted that in 2D models, microglia cells are in a phenotypically different state from their CNS environment: For instance, they often display limited ramifications and higher proliferation with respect to what was found in vivo.

As also reviewed by Watson and coworkers, several hydrogel or fiber-based materials and nanomaterials have been used to culture astrocytes and microglia in 3D in an attempt to achieve more physiologically relevant culture conditions [186]. The BV2 microglia line was grown in the Corning PuraMatrix™ peptide hydrogel. This biomaterial forms a nanometer-scale fibrous structure with an average pore size of 50–200 nm that was shown to support cell proliferation and differentiation. In this matrix, BV2 cells display in vivo–like ramifications demonstrating that the presence of a 3D array of attachment sites can promote such morphology [187]. The presence of ECM proteins seems to interfere with such attachment to a certain extent. In a similar fashion, microglia has been cultured in a 3D collagen matrix [188,189]. Here, it was shown that microglia display ramified phenotype and responds as expected to proinflammatory LPS. Not all biomaterials appear ideal for culturing microglia; in some cases, they can support cells but inherently activate them, too [190]. Remarkably, the matrix biomaterials and microfluidics technologies have been recently integrated towards making models with multiple cell types and a prototype vasculature system [191].

The medium-term perspective of such culture systems is to allow HTS in order to make them useful for drug discovery and testing. A longer-term perspective is the attainment of more complete systems with all the cell types needed to recapitulate the desired section of the CNS. One interesting step in this direction is the use of 3D bioprinting or other manipulation technologies, in order to seed multiple homogeneous groups of cells in a matrix, and let them grow and develop intercellular interactions. Such (pathological or physiological) model systems can be harnessed to study the effects of a large number of drug candidates. In their recent paper, Cai and coworkers showed that acoustic standing waves can drive live cells to cluster in neurospheroids of homogeneous size within a 3D matrix. Cells were manipulated in a contact-less way within a photocrosslinkable matrix of the desired composition that could model a physiological or a pathological state [192]. Different cell types can be mixed while setting up the experiment in order to obtain the desired model system. Interestingly, the authors showed that microglia behave as in vivo when they made neurospheroids with microglia, neurons and Aβ plaques (a model AD state); here, microglia migrated towards the plaques and covered them, as also seen in vivo.

Due to the recent developments in the production of hiPSC, it is now possible to develop long-lived small-scale 3D models of human brain regions. Such 3D cellular models can also contain microglia and be used to characterize the interactions of microglia with neurons and other cells. A breakthrough point in this was the development of protocols by Lancaster and coworkers. These enabled the generation of cerebral organoids from donor’s hiPSC without the use of any inhibitors or molecular pathway manipulators and led to differentiated organoids containing distinct brain regions [193,194]. One of the key procedural differences of this method with respect to the above-described microfluidics or hydrogel-assisted methods was that here a cluster of stem cells was led to develop as an embryoid body first and then differentiated in situ to make different cell lines, instead of mixing a controlled set of differentiated cells. Later, after the embryoid body was differentiated into a cerebral organoid, it was embedded in a Matrigel bead and cultured for extended times in orbital shakers or stirred bioreactors that ensured proper oxygenation and thus viability and development for up to many months. Individual, millimeter-scaled organoids could then be harvested for characterized at the desired growth stage in conditions mimicking physiological or pathological states. It was commonly thought that cells would not differentiate to microglia in such organoids, as they derive from a different germinal layer than neurons. Interestingly, a new avenue was recently opened by Ormel and coworkers, who demonstrated that microglia innately develop within organoids prepared and cultured essentially, as shown by Lancaster [170]. Organoid-grown microglia display the expected inflammatory and phagocytic behavior and transcriptome of mature microglia. Additionally, authors performed super-resolution fluorescence microscopy (STED) and showed the co-localization of microglia processes with post-synaptic neuronal markers. This work proves that the produced organoid model recapitulates the development of brain areas with microglia and also that it can be used as a tool to characterize microglia–neuron interactions during development and in disease. Further progress in this field should lead to automated technologies, to produce large numbers of homogeneous cerebral organoids from hiPSC that can be used in drug development and in a fuller elucidation of the role of microglia in neurodegenerative diseases and of the ways to mitigate such diseases leveraging on that knowledge.

## 7. Microfluidics Technologies for Microglia–Neuron Interaction Studies

The field of microfluidics involves both science and technology of systems that manipulate and process low volumes of fluids (in the range of microliters (10^−6^) to picoliters (10^−12^)), generated in small-scaled cells and microchannels with dimensions of around 100 to 500 mm [195]. Microfluidics technologies have been employed in the field of neurology providing important insights to nervous system research, investigating cellular/molecular mechanisms and complex interactions which occur among neural cells [196]. Conventional methods of analysis, where neurons and glial cells are co-cultured in randomly mixed form are inadequate to maintain biochemical and physiological axon–glia interactions in culture. This approach fails to investigate the localized communication of axons and glia and the processes that distinctively affect various parts of the neuron in neuronal injury and neurodegenerative diseases (e.g., spinal cord injury and Alzheimer’s disease) [197]. On the contrary, microfluidics allows the generation of compartmentalized platforms in which it is possible both to separate the axons from the neuronal cell bodies and, through regulated flows, to investigate interactions between different cell types. In fact, this technology supports culturing of various cell populations in tight proximity to each other, making them an optimal tool for neurons–glia co-culture.

One of the first microfluidic platforms developed for neuronal research was the Campenot chamber, a three-chamber culture system for neuritic isolation [198]. This method provided the division of axons from neuronal cell bodies. Further researches adopting Campenot chamber have contributed to significant discoveries in peripheral nervous system (PNS) axonal development, degeneration and regeneration [199,200]. However, the system required a time-consuming procedure and less than 30% of the devices could be used for experiments. Moreover, the system was unsuitable for live cell imaging, requiring chamber removal and cell fixation [201]. Subsequently, further works led to numerous advances in the field, developing devices with diverse materials like silicon, glass and various elastomers (polydimethylsiloxane (PDMS)), SU8 (negative photoresist) and different design complexities.

Later, several novel platforms allowed to study in depth axonal and neuronal milieu. In particular, microgrooves hosted axon extensions of neurons in proximal contact of the microchannels, providing precise isolation of axons from respective cell bodies [201,202,203]. For example, Taylor et al. published an interesting paper were axonal injury was investigated. The microfluidic culture platform consisted of a PDMS device that was produced by using soft lithography and replica molding. The device design was characterized by a physical barrier with embedded microgrooves separating two specular compartments. The microgrooves allowed only the passage of neuritic processes into the axonal side but not of cell bodies. This conformation provided a modest flow between the compartments that counterbalanced diffusion. This platform was one of the first to coculture oligodendrocytes in the axonal compartment to potentially investigate mechanisms of axonal myelination and demyelination [204].

**Figure 2:**
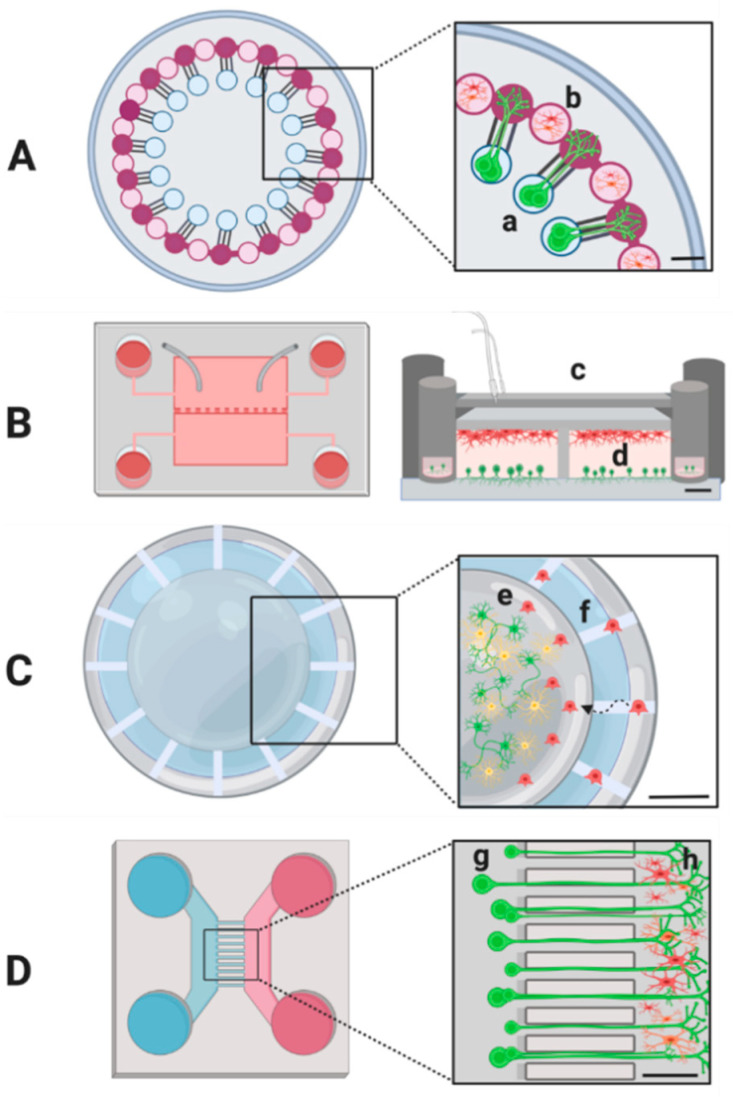
Schematics showing examples of microfluidic platforms from the literature. Microglia (red), neurons (green) and astrocytes (yellow). (**A**) Circular compartmentalized platform with multiple independent units consisting of somal (**a**) and axonal compartments. Microglia is placed in spatially defined areas (**b**) arrayed between bundles of axons outgrowth; scale bar, 500 µm [206]. (**B**) Vertically layered configuration with four mirror image cylinders as media reservoirs with a pressure chamber to control neuron–glia interactions. Cross-section (**c**) showing attached glia on polydimethylsiloxane (PDMS) roof of culture chamber and neurons attached to platform glass surface; scale bar, 200 µm (**d**) [208]. (**C**) 3D tri-culture system consisting of a central matrigel-coated chamber with 3D neurons/astrocytes (**e**) and microglia in angular chambers (**f**). Scale bar, 500 µm [191]. (**D**) Microfluidic platform to establish directional growth and isolation of axons. Microchannels separating somal (**g**) and axonal (**h**) compartments. Scale bar, 250 µm [209].

An interesting work of Hosmane et al. employed a circular microfluidic device for the study of axon–microglia interactions [205]. This geometry has been used for an easier centrifugation and to allow optimal positioning of neurons and glia cells. The developed PDMS device consisted of independent microchambers where microchannels enabled separation of axons and neuronal cell bodies. Additionally, patterned microstenciling was applied to directly place microglia within areas of interest in the axonal compartment (Figure 2A). The authors focused on microglial response to degenerating axons, studying glia migration towards injured axons. Microglia cells were placed in between areas of axonal growth and oxidative stress was induced in the somal compartment. The study revealed a preferential aggregation of microglia to degenerating axons compared to control ones and reported specific microglial responses to axon-derived signals [205]. The same research group used this fabricated device to analyze the mechanisms of microglial clearance of axons by coculturing microglia on the axonal side of the device. In particular, TIR-domain-containing adapter-inducing interferon-β (TRIF) has been found to play a key role in microglial phagocytosis of degenerated axons. Furthermore, the coculture system permitted to analyze microglia activation, studying gene expression related to M1 (iNOS and CD32) and M2 (Arg1 and SRB1) states. This in vitro study combined microfluidic technology to a simultaneous in vivo investigation by performing dorsal root axotomy to TRIF KO mice. The device design modification also provided added value to the present research since they could replicate the in vivo axotomy procedure. In fact, the microfluidic platform was equipped with an open access port to mechanically sever neuron projection to better model axotomy-induced axonal degeneration [206].

Other platforms allow the study of diffusion-based events in reversibly isolated cultures of neurons and glia [207,208]. Majumdar et al. developed a two-cell culture chamber separated through the presence of a microfabricated barrier valve. Chambers connection allowed nutrients exchange and the glia reservoir was maintained higher than that in the neuronal reservoir to improve the flow of media from the glia to the neuronal chamber. The study reported that the presence of glia supported neuronal survival and increased neurons transfection efficiency significantly. Shi et al. created a vertically layered configuration to study neuronal interactions where glia and neurons were co-cultured in a top-bottom disposition with glial cells on the device roofs over the cell chambers, while neurons were cultured on the glass surfaces within the same device (Figure 2B).

As previously anticipated, in order to develop more relevant human brain models, it is possible to combine 3D culture techniques and microfluidics platforms. Park et al. generated a three-dimensional (3D) microfluidic system replicating neurons, astrocytes and microglia interaction in Alzheimer’s disease context. This tri-culture platform was characterized by a central chamber and many peripheral chambers linked to the central one through microchannels. The Matrigel-coated central chamber housed neurons and astrocytes differentiated cells and it was possible to analyze microglia recruitment and phenotype changes via migration channels. This platform application provided valuable advancement over current in vitro human AD models and allowed us to study the human microglia role in neuroinflammatory molecular mechanisms and damages to neurons/astrocytes that could be crucial in AD pathology (Figure 2C) [191].

In 2020, Fujita et al. used a platform where neuronal projections were directed into a confined compartment and exposed to different cellular microenvironments. Specifically, the authors investigated the microglial *NGL1* and neuronal *netrin-G1* signaling that supports microglia accumulation along sub-cerebral projection axons and maintains neuronal survival [209]. Fujita and collaborators used a poly-L-lysine-and-laminin-coated AXIS^TM^ (Axon Isolation Device, Merck; Xona Microfluidics^®^, LLC), a commercial compartmentalized platform which provided fluidic isolation and improved organization over typical disorganized neuronal cell culture (Figure 2D). The technology consisted in a disposable, pre-assembled platform, suitable for human stem-cell-derived neurons providing better cell attachment and long-term growth. The research group further published a protocol describing the procedure for modeling the accumulation of microglia toward neuronal axons, using the abovementioned axon isolation culture device [210].

To conclude, microfluidic platforms have shaped new perspectives on how we handle biological samples and matrices. Microfluidics, compared to conventional macroscale techniques, offer the ability to manipulate cellular microenvironments, to study interactions of distinct molecular agents on individual neurons and glia and allows for the localized analysis of cell-to-cell interactions and single cell projections (axons and dendrites).

## 8. Conclusions

Neuronal and glial cells, including microglia, interact towards the proper brain development and function. This physiological interaction is regulated by a continuous crosstalk between these cells, both through direct cell–cell contact and by release of many different molecules. Interestingly, these communication factors can be released and received by both types of cells, either directly or indirectly, through extracellular vesicles that can transfer proteins, lipids and nucleic acids, especially miRNAs. In particular, factors produced by neurons modulate microglial phenotypes, either maintaining microglia in a surveying phenotype or contributing to their activation towards neuroprotection or neuroinflammation and neurodegeneration. This phenotypic change is driven by a switch in the expression of specific microglial genes, which, in turn, is modulated by epigenetic changes, such as DNA methylation, histone post-translational modifications and activity of miRNAs, that can derive by extracellular vesicles. In all CNS pathological conditions, but especially in chronic neurodegenerative disorders, microglial phenotype is subjected to modifications with disease progression. Activated microglia is neuroprotective in the early stages, while it contributes to neuroinflammation and neurodegeneration in later stages. Even though neurodegenerative diseases include a wide range of pathologies affecting different brains areas, neuronal types and aggregating proteins, all of them share a progressive loss of neuronal properties accompanied by microglia activation and neuroinflammation, and involving an alteration of neuron–microglia crosstalk. The strict interplay between glial cells and neurons is regulated by a huge variety of mechanisms that need to be clarified by using advanced technologies, such as microfluidic, 3D culture and in vivo imaging. The purpose of our review was to recapitulate the most recent studies that provide insight into the complex field of microglia immunomodulation through epigenetic mechanisms and/or released factors corroborating the widespread hypothesis of a diagnostic and therapeutic use of EVs cargo, as well as the promising epigenetic modulation of microglia, to ameliorate disease symptoms.

## Abbreviations

CNS, Central Nervous System; IL, interleukin; ncRNAs, non-coding RNAs; MS, multiple sclerosis; AD, Alzheimer’s disease; PD, Parkinson’s disease; ALS, Amyotrophic lateral sclerosis; TNF, tumor necrosis factor; IFN, interferons; TGF, transforming growth factor; NGF, Nerve Growth Factor; BDNF, Brain-Derived Neurotrophic Factor; iNOS, inducible nitric oxide synthase; IgSF, immunoglobulin superfamily; TREM2, Triggering Receptor Expressed on Myeloid cells-2; DAP12, DNAXactivating protein of 12 kDa; CD, clusters of differentiation; GDNF, glial cell-derived neurotrophic factor; EVs, extracellular vesicles; Hsp, heat-shock proteins; TDP43, TAR DNA-binding protein 43; SOD1, copper zinc superoxide dismutase 1; CB1, type-1 cannabinoid receptors; miRNAs, microRNAs; rRNAs, ribosomal RNA; lncRNAs, long non-coding RNA; tRNAs, transfer RNA; smRNAs, small nuclear RNA; Syt1, presynaptic synaptotagmin1; Nlg1, postsynaptic neuroligin1; NSCs, Neuronal Stem Cells; TBI, Traumatic Brain Injury; APP, amyloid precursor protein; 5mC, cytosine methylation; PSEN1, presenilin 1; HMTs, histone methyltransferases; HDMs, histone demethylases; HATs, histone acetyl transferases; HDACs, histone deacetylases; HTS, high-throughput screening; TSA Trichostatin-A; VPA, valproic acid; Sig-1R, sigma-1 receptor; nSMase2, neural sphingomyelinase 2; hnRNPs, heterogeneous nuclear ribonucleoproteins; miRISC, miRNA induced silencing complex; RSV, Resveratrol; KO, knock-out; WT, wild-type; PRC2, Polycomb repressive complex 2; CSF-1, colony-stimulating factor-1; IVM, Intravital microscopy; MPM, multi-photon microscopy; STED, Stimulated Emission Depletion; STORM, Stochastic optical reconstruction microscopy; PALM, Photoactivated Localization Microscopy; SIM, structured illumination microscopy; EM, electron microscopy; CLEM, correlative light and electron microscopy; TEM, Transmission Electron Microscopy; STEM, scanning transmission electron microscopy; STED, super-resolution fluorescence microscopy; PNS, peripheral nervous system; PDMS, polydimethylsiloxane; TRIF, TIR-domain-containing adapter-inducing interferon.

## References

[1] Kettenmann H., Hanisch U.K., Noda M., Verkhratsky A. (2011). Physiology of microglia. Physiol. Rev..

[2] Cuadros M.A., Martin C., Coltey P., Almendros A., Navascués J. (1993). First appearance, distribution, and origin of macrophages in the early development of the avian central nervous system. J. Comp. Neurol..

[3] Ginhoux F., Greter M., Leboeuf M., Nandi S., See P., Gokhan S., Mehler M.F., Conway S.J., Ng L.G., Stanley E.R. (2010). Fate mapping analysis reveals that adult microglia derive from primitive macrophages. Science.

[4] Nimmerjahn A., Kirchhoff F., Helmchen F. (2005). Resting microglial cells are highly dynamic surveillants of brain parenchyma in vivo. Science.

[5] Xu Y., Xu Y., Wang Y., Wang Y., He L., Jiang Z., Huang Z., Liao H., Li J., Saavedra J.M. (2015). Telmisartan prevention of LPS-induced microglia activation involves M2 microglia polarization via CaMKKβ-dependent AMPK activation. Brain Behav. Immun..

[6] Lisi L., Ciotti G.M., Braun D., Kalinin S., Currò D., Russo C.D., Coli A., Mangiola A., Anile C., Feinstein D.L. (2017). Expression of iNOS, CD163 and ARG-1 taken as M1 and M2 markers of microglial polarization in human glioblastoma and the surrounding normal parenchyma. Neurosci. Lett..

[7] Meng X., Duan C., Pang H., Chen Q., Han B., Zha C., Dinislam M., Wu P., Li Z., Zhao S. (2019). DNA damage repair alterations modulate M2 polarization of microglia to remodel the tumor microenvironment via the p53-mediated MDK expression in glioma. EBioMedicine.

[8] Chamera K., Trojan E., Szuster-Głuszczak M., Basta-Kaim A. (2020). The Potential Role of Dysfunctions in Neuron-Microglia Communication in the Pathogenesis of Brain Disorders. Curr. Neuropharmacol..

[9] Wake H., Moorhouse A.J., Nabekura J. (2011). Functions of microglia in the central nervous system--beyond the immune response. Neuron Glia Biol..

[10] Eyo U.B., Wu L.J. (2013). Bidirectional microglia-neuron communication in the healthy brain. Neural Plast..

[11] Marinelli S., Basilico B., Marrone M.C., Ragozzino D. (2019). Microglia-neuron crosstalk: Signaling mechanism and control of synaptic transmission. Semin. Cell Dev. Biol..

[12] Zhang L., Xu J., Gao J., Wu Y., Yin M., Zhao W. (2018). CD200-, CX3CL1-, and TREM2-mediated neuron-microglia interactions and their involvements in Alzheimer’s disease. Rev. Neurosci..

[13] Sato K. (2015). Effects of Microglia on Neurogenesis. Glia.

[14] Šišková Z., Tremblay M.È. (2013). Microglia and synapse: Interactions in health and neurodegeneration. Neural Plast..

[15] Wu Y., Dissing-Olesen L., MacVicar B.A., Stevens B. (2015). Microglia: Dynamic Mediators of Synapse Development and Plasticity. Trends Immunol..

[16] Wake H., Moorhouse A.J., Miyamoto A., Nabekura J. (2013). Microglia: Actively surveying and shaping neuronal circuit structure and function. Trends Neurosci..

[17] Deverman B.E., Patterson P.H. (2009). Cytokines and CNS development. Neuron.

[18] Kerschensteiner M., Meinl E., Hohlfeld R. (2009). Neuro-immune crosstalk in CNS diseases. Neuroscience.

[19] Smith J.A., Das A., Ray S.K., Banik N.L. (2012). Role of pro-inflammatory cytokines released from microglia in neurodegenerative diseases. Brain Res. Bull..

[20] Biber K., Neumann H., Inoue K., Boddeke H.W. (2007). Neuronal ‘On’ and ‘Off’ signals control microglia. Trends Neurosci..

[21] Hanisch U.K. (2002). Microglia as a source and target of cytokines. Glia.

[22] Fixe P., Praloran V. (1998). M-CSF: Haematopoietic growth factor or inflammatory cytokine?. Cytokine.

[23] Wyss-Coray T., Lin C., Yan F., Yu G.Q., Rohde M., McConlogue L., Masliah E., Mucke L. (2001). TGF-beta1 promotes microglial amyloid beta clearance and reduces plaque burden in transgenic mice. Nat. Med..

[24] Suzumura A. (2014). Microglia in neurodegenerative disorders and neuroinflammation. Rinsho Shinkeigaku.

[25] Akiyama H., Barger S., Barnum S., Bradt B., Bauer J., Cole G.M., Cooper N.R., Eikelenboom P., Emmerling M., Fiebich B.L. (2000). Inflammation and Alzheimer’s disease. Neurobiol. Aging.

[26] McGeer P.L., McGeer E.G. (2001). Inflammation, autotoxicity and Alzheimer disease. Neurobiol. Aging.

[27] Parajuli B., Horiuchi H., Mizuno T., Takeuchi H., Suzumura A. (2015). CCL11 enhances excitotoxic neuronal death by producing reactive oxygen species in microglia. Glia.

[28] Feng C., Wang X., Liu T., Zhang M., Xu G., Ni Y. (2017). Expression of CCL2 and its receptor in activation and migration of microglia and monocytes induced by photoreceptor apoptosis. Mol. Vis..

[29] Ransohoff R.M., El Khoury J. (2015). Microglia in Health and Disease. Cold Spring Harb. Perspect. Biol..

[30] Paolicelli R.C., Bisht K., Tremblay M.E. (2014). Fractalkine regulation of microglial physiology and consequences on the brain and behavior. Front. Cell Neurosci..

[31] Zujovic V., Benavides J., Vige X., Carter C., Taupin V. (2000). Fractalkine modulates TNF-alpha secretion and neurotoxicity induced by microglial activation. Glia.

[32] Gemma C., Bachstetter A.D., Bickford P.C. (2010). Neuron-Microglia Dialogue and Hippocampal Neurogenesis in the Aged Brain. Aging Dis..

[33] Rogers J.T., Morganti J.M., Bachstetter A.D., Hudson C.E., Peters M.M., Grimmig B.A., Weeber E.J., Bickford P.C., Gemma C. (2011). CX3CR1 deficiency leads to impairment of hippocampal cognitive function and synaptic plasticity. J. Neurosci..

[34] Cardona A.E. (2006). Control of microglial neurotoxicity by the fractalkine receptor. Nat. Neurosci..

[35] Uyemura K., Asou H., Yazaki T., Takeda Y. (1996). Cell-adhesion proteins of the immunoglobulin superfamily in the nervous system. Essays Biochem..

[36] Jiang T., Tan L., Zhu X.C., Zhang Q.Q., Cao L., Tan M.S., Gus L.Z., Wang H.F., Ding Z.Z., Zhang Y.D. (2014). Upregulation of TREM2 ameliorates neuropathology and rescues spatial cognitive impairment in a transgenic mouse model of Alzheimer’s disease. Neuropsychopharmacology.

[37] Painter M.M., Atagi Y., Liu C.C., Rademakers R., Xu H., Fryer J.D., Bu G. (2015). TREM2 in CNS homeostasis and neurodegenerative disease. Mol. Neurodegener..

[38] Edmonson C., Ziats M.N., Rennert O.M. (2014). Altered glial marker expression in autistic post-mortem prefrontal cortex and cerebellum. Mol. Autism..

[39] Filipello F., Morini R., Corradini I., Zerbi V., Canzi A., Michalski B., Erreni M., Markicevic M., Starvaggi-Cucuzza C., Otero K. (2018). The microglial innate immune receptor TREM2 is required for synapse elimination and normal brain connectivity. Immunity.

[40] Walker D.G., Lue L.F. (2015). Immune phenotypes of microglia in human neurodegenerative disease: Challenges to detecting microglial polarization in human brains. Alzheimers Res. Ther..

[41] Lyons A., Downer E.J., Crotty S., Nolan Y.M., Mills K.H.G., Lynch M.A. (2007). CD200 ligand-receptor interaction modulates microglial activation in vivo and in vitro: A role for IL-4. J. Neurosci..

[42] Lyons A., McQuillan K., Deighan B.F., O’Reilly J.A., Downer E.J., Murphy A.C., Watson M., Piazza A., O’Connell F., Griffin R. (2009). Decreased neuronal CD200 expression in IL-4-deficient mice results in increased neuroinflammation in response to lipopolysaccharide. Brain Behav. Immun..

[43] Trotta T., Panaro M.A., Cianciulli A., Mori G., Di Benedetto A., Porro C. (2018). Microglia-derived extracellular vesicles in Alzheimer’s Disease: A double-edged sword. Biochem. Pharmacol..

[44] Zhang Y., Liu Y., Liu H., Tang W.H. (2019). Exosomes: Biogenesis, biologic function and clinical potential. Cell Biosci..

[45] Losurdo M., Pedrazzoli M., D’Agostino C., Elia C.A., Massenzio F., Lonati E., Mauri M., Rizzi L., Molteni L., Bresciani E. (2020). Intranasal delivery of mesenchymal stem cell-derived extracellular vesicles exerts immunomodulatory and neuroprotective effects in a 3xTg model of Alzheimer’s disease. Stem Cells Transl. Med..

[46] Budnik V., Ruiz-Cañada C., Wendler F. (2016). Extracellular vesicles round off communication in the nervous system. Nat. Rev. Neurosci..

[47] De Haas A.H., Van Weering H.R.J., De Jong E.K., Boddeke H.W.G.M., Biber K.P.H. (2007). Neuronal chemokines: Versatile messengers in central nervous system cell interaction. Mol. Neurobiol..

[48] Yoon Y.J., Kim O.Y., Gho Y.S. (2014). Extracellular vesicles as emerging intercellular communicasomes. BMB Rep..

[49] Glebov K., Löchner M., Jabs R., Lau T., Merkel O., Schloss P., Steinhäuser C., Walter J. (2015). Serotonin stimulates secretion of exosomes from microglia cells. Glia.

[50] Schneider A., Simons M. (2013). Exosomes: Vesicular carriers for intercellular communication in neurodegenerative disorders. Cell Tissue Res..

[51] Vingtdeux V., Sergeant N., Buee L. (2012). Potential contribution of exosomes to the prion-like propagation of lesions in Alzheimer’s disease. Front. Physiol..

[52] Antonucci F., Turola E., Riganti L., Caleo M., Gabrielli M., Perrotta C., Novellino L., Clementi E., Giussani P., Viani P. (2012). Microvesicles released from microglia stimulate synaptic activity via enhanced sphingolipid metabolism. EMBO J..

[53] Gabrielli M., Battista N., Riganti L., Prada I., Antonucci F., Cantone L., Matteoli M., Maccarrone M., Verderio C. (2015). Active endocannabinoids are secreted on extracellular membrane vesicles. EMBO Rep..

[54] Kalluri R., LeBleu V.S. (2016). Discovery of Double-Stranded Genomic DNA in Circulating Exosomes. Cold Spring Harb. Symp. Quant. Biol..

[55] Etheridge A., Lee I., Hood L., Galas D., Wang K. (2011). Extracellular microRNA: A new source of biomarkers. Mutat. Res..

[56] Alexander M., Hu R., Runtsch M.C., Kagele D.A., Mosbruger T.L., Tolmachova T., Seabra M.C., Round J.L., Ward D.M., O’Connell R.M. (2015). Exosome-delivered microRNAs modulate the inflammatory response to endotoxin. Nat. Commun..

[57] Lemaire Q., Raffo-Romero A., Arab T., Van Camp C., Drago F., Forte S., Gimeno J.P., Begard S., Colin M., Vizioli J. (2019). Isolation of microglia-derived extracellular vesicles: Towards miRNA signatures and neuroprotection. J. Nanobio Technol..

[58] Prada I., Gabrielli M., Turola E., Iorio A., D’Arrigo G., Parolisi R., De Luca M., Pacifici M., Bastoni M., Lombardi M. (2018). Glia-to-neuron transfer of miRNAs via extracellular vesicles: A new mechanism underlying inflammation-induced synaptic alterations. Acta Neuropathol..

[59] Morton M.C., Neckles V.N., Seluzicki C.M., Holmberg J.C., Feliciano D.M. (2018). Neonatal Subventricular Zone Neural Stem Cells Release Extracellular Vesicles that Act as a Microglial Morphogen. Cell Rep..

[60] Pinto S., Cunha C., Barbosa M., Vaz A.R., Brites D. (2017). Exosomes from NSC-34 Cells Transfected with hSOD1-G93A are Enriched in miR-124 and Drive Alterations in Microglia Phenotype. Front. Neurosci..

[61] Song Y., Li Z., He T., Qu M., Jiang L., Li W., Shi X., Pan J., Zhang L., Wang Y. (2019). M2 microglia-derived exosomes protect the mouse brain from ischemia-reperfusion injury via exosomal miR-124. Theranostics.

[62] Sohel M.H. (2016). Extracellular/Circulating MicroRNAs: Release Mechanisms, Functions and Challenges. Achiev. Life Sci..

[63] Akers J.C., Ramakrishnan V., Kim R., Skog J., Nakano I., Pingle S., Kalinina J., Hua W., Kesari S., Mao Y. (2013). MiR-21 in the extracellular vesicles (EVs) of cerebrospinal fluid (CSF): A platform for glioblastoma biomarker development. PLoS ONE.

[64] Zhang L., Dong L.Y., Li Y.J., Hong Z., Wei W.S. (2012). The microRNA miR-181c controls microglia-mediated neuronal apoptosis by suppressing tumor necrosis factor. J. Neuroinflamm..

[65] Vaz A.R., Pinto S., Ezequiel C., Cunha C., Carvalho L.A., Moreira R., Brites D. (2019). Phenotypic Effects of Wild-Type and Mutant SOD1 Expression in N9 Murine Microglia at Steady State, Inflammatory and Immunomodulatory Conditions. Front. Cell Neurosci..

[66] Zhang Y., Xu C., Nan Y., Nan S. (2020). Microglia-Derived Extracellular Vesicles Carrying miR-711 Alleviate Neurodegeneration in a Murine Alzheimer’s Disease Model by Binding to Itpkb. Front. Cell Dev. Biol..

[67] Chen O., Donnelly C.R., Ji R.R. (2020). Regulation of pain by neuro-immune interactions between macrophages and nociceptor sensory neurons. Curr. Opin. Neurobiol..

[68] Fernandes A., Ribeiro A.R., Monteiro M., Garcia G., Vaz A.R., Brites D. (2018). Secretome from SH-SY5Y APP Swe cells trigger time-dependent CHME3 microglia activation phenotypes, ultimately leading to miR-21 exosome shuttling. Biochimie.

[69] Ge X., Guo M., Hu T., Li W., Huang S., Yin Z., Zhang J. (2020). Increased Microglial Exosomal miR-124-3p Alleviates Neurodegeneration and Improves Cognitive Outcome after rmTBI. Mol. Ther..

[70] Han C., Guo L., Yang Y., Guan Q., Shen H., Sheng Y., Jiao Q. (2020). Mechanism of microRNA-22 in regulating neuroinflammation in Alzheimer’s disease. Brain Behav..

[71] Esteller M. (2008). Epigenetics in cancer. N. Engl. J. Med..

[72] Bradley-Whitman M.A., Lovell M.A. (2013). Epigenetic changes in the progression of Alzheimer’s disease. Mech. Aging Dev..

[73] Coppieters N., Dieriks B.V., Lill C., Faull R.L., Curtis M.A., Dragunow M. (2014). Global changes in DNA methylation and hydroxymethylation in Alzheimer’s disease human brain. Neurobiol. Aging.

[74] Day J.J., Sweatt J.D. (2011). Cognitive neuroepigenetics: A role for epigenetic mechanisms in learning and memory. Neurobiol. Learn Mem..

[75] Pang K.K.L., Sharma M., Sajikumar S. (2019). Epigenetics and memory: Emerging role of histone lysine methyltransferase G9a/GLP complex as bidirectional regulator of synaptic plasticity. Neurobiol. Learn Mem..

[76] Cho S.H., Chen J.A., Sayed F., Ward M.E., Gao F., Nguyen T.A., Krabbe G., Sohn P.D., Lo I., Minami S. (2015). SIRT1 deficiency in microglia contributes to cognitive decline in aging and neurodegeneration via epigenetic regulation of IL-1beta. J. Neurosci..

[77] Matt S.M., Lawson M.A., Johnson R.W. (2016). Aging and peripheral lipopolysaccharide can modulate epigenetic regulators and decrease IL-1β promoter DNA methylation in microglia. Neurobiol. Aging.

[78] Bakulski K.M., Dolinoy D.C., Sartor M.A., Paulson H.L., Konen J.R., Lieberman A.P., Albin R.L., Hu H., Rozek L.S. (2012). Genome-wide DNA methylation differences between late-onset Alzheimer’s disease and cognitively normal controls in human frontal cortex. J. Alzheimers Dis..

[79] Mastroeni D., Grover A., Delvaux E., Whiteside C., Coleman P.D., Rogers J. (2010). Epigenetic changes in Alzheimer’s disease: Decrements in DNA methylation. Neurobiol. Aging..

[80] Chouliaras L., Mastroeni D., Delvaux E., Grover A., Kenis G., Hof P.R., Steinbusch H.W., Coleman P.D., Rutten B.P., van den Hove D.L. (2013). Consistent decrease in global DNA methylation and hydroxymethylation in the hippocampus of Alzheimer’s disease patients. Neurobiol. Aging.

[81] Sathasivam K., Neueder A., Gipson T.A., Landles C., Benjamin A.C., Bondulich M.K., Smith D.L., Faull R.L., Roos R.A., Howland D. (2013). Aberrant splicing of HTT generates the pathogenic exon 1 protein in Huntington disease. Proc. Natl. Acad. Sci. USA.

[82] Sanchez-Mut J.V., Heyn H., Vidal E., Moran S., Sayols S., Delgado-Morales R., Schultz M.D., Ansoleaga B., Garcia-Esparcia P., Pons-Espinal M. (2016). Human DNA methylomes of neurodegenerative diseases show common epigenomic patterns. Transl. Psychiatry.

[83] Gijselinck I., Van Mossevelde S., van der Zee J., Sieben A., Engelborghs S., De Bleecker J., Ivanoiu A., Deryck O., Edbauer D., Zhang M. (2016). The C9orf72 repeat size correlates with onset age of disease, DNA methylation and transcriptional downregulation of the promoter. Mol. Psychiatry.

[84] Lin H.C., Hsieh H.M., Chen Y.H., Hu M.L. (2009). S-Adenosylhomocysteine increases beta-amyloid formation in BV-2 microglial cells by increased expressions of beta-amyloid precursor protein and presenilin 1 and by hypomethylation of these gene promoters. Neurotoxicology.

[85] Byun C.J., Seo J., Jo S.A., Park Y.J., Klug M., Rehli M., Park M.H., Jo I. (2012). DNA methylation of the 5′-untranslated region at +298 and +351 represses BACE1 expression in mouse BV-2 microglial cells. Biochem. Biophys. Res. Commun..

[86] Moussa-Pacha N.M., Abdin S.M., Omar H.A., Alniss H., Al-Tel T.H. (2020). BACE1 inhibitors: Current status and future directions in treating Alzheimer’s disease. Med. Res. Rev..

[87] Prati F., De Simone A., Armirotti A., Summa M., Pizzirani D., Scarpelli R., Bertozzi S.M., Perez D.I., Andrisano V., Perez-Castillo A. (2015). 3,4-Dihydro-1,3,5-triazin-2(1H)-ones as the First Dual BACE-1/GSK-3β Fragment Hits against Alzheimer’s Disease. ACS Chem. Neurosci..

[88] Griñán-Ferré C., Marsal-García L., Bellver-Sanchis A., Kondengaden S.M., Turga R.C., Vázquez S., Pallàs M. (2019). Pharmacological inhibition of G9a/GLP restores cognition and reduces oxidative stress, neuroinflammation and β-Amyloid plaques in an early-onset Alzheimer’s disease mouse model. Aging.

[89] Kelly R.D., Cowley S.M. (2013). The physiological roles of histone deacetylase (HDAC) 1 and 2: Complex co-stars with multiple leading parts. Biochem. Soc. Trans..

[90] Datta M., Staszewski O., Raschi E., Frosch M., Hagemeyer N., Tay T.L., Blank T., Kreutzfeldt M., Merkler D., Ziegler-Waldkirch S. (2018). Histone deacetylases 1 and 2 regulate microglia function during development, homeostasis, and neurodegeneration in a context-dependent manner. Immunity.

[91] Buonvicino D., Felici R., Ranieri G., Caramelli R., Lapucci A., Cavone L., Muzzi M., Di Pietro L., Bernardini C., Zwergel C. (2018). Effects of Class II-Selective Histone Deacetylase Inhibitor on Neuromuscular Function and Disease Progression in SOD1-ALS Mice. Neuroscience.

[92] Harrison I.F., Crum W.R., Vernon A.C., Dexter D.T. (2015). Neurorestoration induced by the HDAC inhibitor sodium valproate in the lactacystin model of Parkinson’s is associated with histone acetylation and up-regulation of neurotrophic factors. Br. J. Pharmacol..

[93] Kim T., Song S., Park Y., Kang S., Seo H. (2019). HDAC Inhibition by Valproic Acid Induces Neuroprotection and Improvement of PD-like Behaviors in LRRK2 R1441G Transgenic Mice. Exp. Neurobiol..

[94] Monti B., Gatta V., Piretti F., Raffaelli S.S., Virgili M., Contestabile A. (2010). Valproic acid is neuroprotective in the rotenone rat model of Parkinson’s disease: Involvement of alpha-synuclein. Neurotox. Res..

[95] Romeiro L.A.S., da Nunes J.L.C., de Miranda C.O., Cardoso G.S.H.R., de Oliveira A.S., Gandini A., Kobrlova T., Soukup O., Rossi M., Senger J. (2019). Novel Sustainable-by-Design HDAC Inhibitors for the Treatment of Alzheimer’s Disease. ACS Med. Chem. Lett..

[96] Han S.B., Lee J.K. (2009). Anti-inflammatory effect of Trichostatin-A on murine bone marrow-derived macrophages. Arch. Pharm. Res..

[97] Kannan V., Brouwer N., Hanisch U.K., Regen T., Eggen B.J., Boddeke H.W. (2013). Histone deacetylase inhibitors suppress immune activation in primary mouse microglia. J. Neurosci. Res..

[98] Lin F.L., Yen J.L., Kuo Y.C., Kang J.J., Cheng Y.W., Huang W.J., Hsiao G. (2019). HADC8 Inhibitor WK2-16 Therapeutically Targets Lipopolysaccharide-Induced Mouse Model of Neuroinflammation and Microglial Activation. Int. J. Mol. Sci..

[99] Lee H.Y., Fan S.J., Huang F.I., Chao H.Y., Hsu K.C., Lin T.E., Yeh T.K., Lai M.J., Li Y.H., Huang H.L. (2018). 5-Aroylindoles Act as Selective Histone Deacetylase 6 Inhibitors Ameliorating Alzheimer’s Disease Phenotypes. J. Med. Chem..

[100] Gal J., Chen J., Barnett K.R., Yang L., Brumley E., Zhu H. (2013). HDAC6 regulates mutant SOD1 aggregation through two SMIR motifs and tubulin acetylation. J. Biol. Chem..

[101] Taes I., Timmers M., Hersmus N., Bento-Abreu A., Van Den Bosch L., Van Damme P., Robberecht W. (2013). Hdac6 deletion delays disease progression in the SOD1G93A mouse model of ALS. Hum. Mol. Genet..

[102] Guo W., Naujock M., Fumagalli L., Vandoorne T., Baatsen P., Boon R., Ordovás L., Patel A., Welters M., Vanwelden T. (2017). HDAC6 inhibition reverses axonal transport defects in motor neurons derived from FUS-ALS patients. Nat. Commun..

[103] Iwamoto M., Nakamura Y., Takemura M., Hisaoka-Nakashima K., Morioka N. (2020). TLR4-TAK1-p38 MAPK pathway and HDAC6 regulate the expression of sigma-1 receptors in rat primary cultured microglia. J. Pharmacol. Sci..

[104] Chao J., Zhang Y., Du L., Zhou R., Wu X., Shen K., Yao H. (2017). Molecular mechanisms underlying the involvement of the sigma-1 receptor in methamphetamine-mediated microglial polarization. Sci. Rep..

[105] Jiao F.Z., Wang Y., Zhang H.Y., Zhang W.B., Wang L.W., Gong Z.J. (2018). Histone Deacetylase 2 Inhibitor CAY10683 Alleviates Lipopolysaccharide Induced Neuroinflammation Through Attenuating TLR4/NF-κB Signaling Pathway. Neurochem. Res..

[106] Chen P.S., Peng G.S., Li G., Yang S., Wu X., Wang C.C., Wilson B., Lu R.B., Gean P.W., Chuang D.M. (2006). Valproate protects dopaminergic neurons in midbrain neuron/glia cultures by stimulating the release of neurotrophic factors from astrocytes. Mol. Psychiatry.

[107] Harrison I.F., Smith A.D., Dexter D.T. (2018). Pathological histone acetylation in Parkinson’s disease: Neuroprotection and inhibition of microglial activation through SIRT 2 inhibition. Neurosci. Lett..

[108] Peng G.S., Li G., Tzeng N.S., Chen P.S., Chuang D.M., Hsu Y.D., Yang S., Hong J.S. (2005). Valproate pretreatment protects dopaminergic neurons from LPS-induced neurotoxicity in rat primary midbrain cultures: Role of microglia. Brain Res. Mol. Brain Res..

[109] Wang P., Zhang Y., Gong Y., Yang R., Chen Z., Hu W., Wu Y., Gao M., Xu X., Qin Y. (2018). Sodium butyrate triggers a functional elongation of microglial process via Akt-small RhoGTPase activation and HDACs inhibition. Neurobiol. Dis..

[110] Slota J.A., Booth S.A. (2019). MicroRNAs in Neuroinflammation: Implications in Disease Pathogenesis, Biomarker Discovery and Therapeutic Applications. Noncoding RNA.

[111] Jonas S., Izaurralde E. (2015). Towards a molecular understanding of microRNA-mediated gene silencing. Nat. Rev. Genet..

[112] Gulyaeva L.F., Kushlinskiy N.E. (2016). Regulatory mechanisms of microRNA expression. J. Transl. Med..

[113] Paolicelli R.C., Bergamini G., Rajendran L. (2019). Cell-to-cell Communication by Extracellular Vesicles: Focus on Microglia. Neuroscience.

[114] Zhang J., Li S., Li L., Li M., Guo C., Yao J., Mi S. (2015). Exosome and Exosomal MicroRNA: Trafficking, Sorting, and Function, Genomics, Proteomics. Bioinformatics.

[115] Freilich R.W., Woodbury M.E., Ikezu T. (2013). Integrated expression profiles of mRNA and miRNA in polarized primary murine microglia. PLoS ONE.

[116] Cardoso A.L., Guedes J.R., de Almeida L.P., de Lima M.C.P. (2012). miR-155 modulates microglia-mediated immune response by down-regulating SOCS-1 and promoting cytokine and nitric oxide production. Immunology.

[117] Martin N.A., Hyrlov K.H., Elkjaer M.L., Thygesen E.K., Wlodarczyk A., Elbaek K.J., Aboo C., Okarmus J., Benedikz E., Reynolds R. (2020). Absence of miRNA-146a Differentially Alters Microglia Function and Proteome. Front. Immunol..

[118] Tahamtan A., Teymoori-Rad M., Nakstad B., Salimi V. (2018). Anti-Inflammatory MicroRNAs and Their Potential for Inflammatory Diseases Treatment. Front. Immunol..

[119] Vergadi E., Vaporidi K., Theodorakis E.E., Doxaki C., Lagoudaki E., Ieronymaki E., Alexaki V.I., Helms M., Kondili E., Soennichsen B. (2014). Akt2 deficiency protects from acute lung injury via alternative macrophage activation and miR-146a induction in mice. J. Immunol..

[120] Wang L., Zhao H., Wang L., Tao Y., Du G., Guan W., Liu J., Brennan C., Ho C.T., Li S. (2020). Effects of Selected Resveratrol Analogues on Activation and Polarization of Lipopolysaccharide-Stimulated BV-2 Microglial Cells. J. Agric. Food Chem..

[121] Ge Y.T., Zhong A.Q., Xu G.F., Lu Y. (2019). Resveratrol protects BV2 mouse microglial cells against LPS-induced inflammatory injury by altering the miR-146a-5p/TRAF6/NF-κB axis. Immun. Pharmacol. Immunotoxicol..

[122] Juknat A., Gao F., Coppola G., Vogel Z., Kozela E. (2019). miRNA expression profiles and molecular networks in resting and LPS-activated BV-2 microglia-Effect of cannabinoids. PLoS ONE.

[123] Gao F., Shen J., Zhao L., Hao Q., Yang Y. (2019). Curcumin Alleviates Lipopolysaccharide (LPS)-Activated Neuroinflammation via Modulation of miR-199b-5p/IκB Kinase β (IKKβ)/Nuclear Factor Kappa B (NF-κB) Pathway in Microglia. Med. Sci. Monit..

[124] Parisi C., Napoli G., Amadio S., Spalloni A., Apolloni S., Longone P., Volonté C. (2016). MicroRNA-125b regulates microglia activation and motor neuron death in ALS. Cell Death Differ..

[125] Aga M., Watters J.J., Pfeiffer Z.A., Wiepz G.J., Sommer J.A., Bertics P.J. (2004). Evidence for nucleotide receptor modulation of cross talk between MAP kinase and NF-kappa B signaling pathways in murine RAW 264.7 macrophages. Am. J. Physiol. Cell Physiol..

[126] Ferrari D., Wesselborg S., Bauer M.K., Schulze-Osthoff K. (1997). Extracellular ATP activates transcription factor NF-kappaB through the P2Z purinoreceptor by selectively targeting NF-kappaB p65. J. Cell Biol..

[127] Parisi C., Arisi I., D’Ambrosi N., Storti A.E., Brandi R., D’Onofrio M., Volonté C. (2013). Dysregulated microRNAs in amyotrophic lateral sclerosis microglia modulate genes linked to neuroinflammation. Cell Death Dis..

[128] Veremeyko T., Yung A.W.Y., Dukhinova M., Strekalova T., Ponomarev E.D. (2019). The Role of Neuronal Factors in the Epigenetic Reprogramming of Microglia in the Normal and Diseased Central Nervous System. Front. Cell Neurosci..

[129] Ayata P., Badimon A., Strasburger H.J., Duff M.K., Montgomery S.E., Loh Y.E., Ebert A., Pimenova A.A., Ramirez B.R., Chan A.T. (2018). Epigenetic regulation of brain region-specific microglia clearance activity. Nat. Neurosci..

[130] Margueron R., Reinberg D. (2011). The Polycomb complex PRC2 and its mark in life. Nature.

[131] Swigut T., Wysocka J. (2007). H3K27 Demethylases, at Long Last. Cell.

[132] Ponomarev E.D., Maresz K., Tan Y., Dittel B.N. (2007). CNS-derived interleukin-4 is essential for the regulation of autoimmune inflammation and induces a state of alternative activation in microglial cells. J. Neurosci..

[133] Li Q., Barres B.A. (2018). Microglia and macrophages in brain homeostasis and disease. Nat. Rev. Immunol..

[134] Elmore M.R.P., Najafi A.R., Koike M.A., Dagher N.N., Spangenberg E.E., Rice R.A., Kitazawa M., Matusow B., Nguyen H., West B.L. (2014). Colony-stimulating factor 1 receptor signaling is necessary for microglia viability, unmasking a microglia progenitor cell in the adult brain. Neuron.

[135] Fourgeaud L., Través P.G., Tufail Y., Leal-Bailey H., Lew E.D., Burrola P.G., Callaway P., Zagórska A., Rothlin C.V., Nimmerjahn A. (2016). TAM receptors regulate multiple features of microglial physiology. Nature.

[136] Goldmann T., Wieghofer P., Jordão M.J.C., Prutek F., Hagemeyer N., Frenzel K., Amann L., Staszewski O., Kierdorf K., Krueger M. (2016). Origin, fate and dynamics of macrophages at central nervous system interfaces. Nat. Immunol..

[137] Rothhammer V., Borucki D.M., Tjon E.C., Takenaka M.C., Chao C.C., Ardura-Fabregat A., de Lima K.A., Gutiérrez-Vázquez C., Hewson P., Staszewski O. (2018). Microglial control of astrocytes in response to microbial metabolites. Nature.

[138] Gosselin D., Link V.M., Romanoski C.E., Fonseca G.J., Eichenfield D.Z., Spann N.J., Stender J.D., Chun H.B., Garner H., Geissmann F. (2014). Environment drives selection and function of enhancers controlling tissue-specific macrophage identities. Cell.

[139] Holtman I.R., Skola D., Glass C.K. (2017). Transcriptional control of microglia phenotypes in health and disease. J. Clin. Investig..

[140] Cheray M., Joseph B. (2018). Epigenetics Control Microglia Plasticity. Front. Cell Neurosci..

[141] de Groot A.E., Pienta K.J. (2018). Epigenetic control of macrophage polarization: Implications for targeting tumor-associated macrophages. Oncotarget.

[142] Zhao L., Zabel M.K., Wang X., Ma W., Shah P., Fariss R.N., Qian H., Parkhurst C.N., Gan W.B., Wong W.T. (2015). Microglial phagocytosis of living photoreceptors contributes to inherited retinal degeneration. EMBO Mol. Med..

[143] Tikhanovich I., Zhao J., Olson J., Adams A., Taylor R., Bridges B., Marshall L., Roberts B., Weinman S.A. (2017). Protein arginine methyltransferase 1 modulates innate immune responses through regulation of peroxisome proliferator-activated receptor γ-dependent macrophage differentiation. J. Biol. Chem..

[144] Kittan N.A., Allen R.M., Dhaliwal A., Cavassani K.A., Schaller M., Gallagher K.A., Carson W.F., Mukherjee S., Grembecka J., Cierpicki T. (2013). Cytokine induced phenotypic and epigenetic signatures are key to establishing specific macrophage phenotypes. PLoS ONE.

[145] Tang Y., Li T., Li J., Yang J., Liu H., Zhang X.J., Le W. (2014). Jmjd3 is essential for the epigenetic modulation of microglia phenotypes in the immune pathogenesis of Parkinson’s disease. Cell Death Differ..

[146] Carrier M., Robert M.È., Ibáñez F.G., Desjardins M., Tremblay M.È. (2020). Imaging the Neuroimmune Dynamics Across Space and Time. Front. Neurosci..

[147] Gabriel E.M., Fisher D.T., Evans S., Takabe K., Skitzki J.J. (2018). Intravital microscopy in the study of the tumor microenvironment: From bench to human application. Oncotarget.

[148] Misgeld T., Kerschensteiner M. (2006). In vivo imaging of the diseased nervous system. Nat. Rev. Neurosci..

[149] Pittet M.J., Weissleder R. (2011). Intravital imaging. Cell.

[150] Prunier C., Chen N., Ritsma L., Vrisekoop N. (2017). Procedures and applications of long-term intravital microscopy. Methods.

[151] Weigert R., Sramkova M., Parente L., Amornphimoltham P., Masedunskas A. (2010). Intravital microscopy: A novel tool to study cell biology in living animals. Histochem. Cell Biol..

[152] Lana D., Ugolini F., Giovannini M.G. (2020). An Overview on the Differential Interplay among Neurons–Astrocytes–Microglia in CA1 and CA3 Hippocampus in Hypoxia/Ischemia. Front. Cell Neurosci..

[153] Mittal K., Eremenko E., Berner O., Elyahu Y., Strominger I., Apelblat D., Nemirovsky A., Spiegel I., Monsonego A. (2019). CD4 T Cells Induce A Subset of MHCII-Expressing Microglia that Attenuates Alzheimer Pathology. Science.

[154] Otxoa-de-Amezaga A., Miró-Mur F., Pedragosa J., Gallizioli M., Justicia C., Gaja-Capdevila N., Ruíz-Jaen F., Salas-Perdomo A., Bosch A., Calvo M. (2019). Microglial cell loss after ischemic stroke favors brain neutrophil accumulation. Acta Neuropathol..

[155] Graf B.W., Boppart S.A. (2010). Imaging and analysis of three-dimensional cell culture models. Methods Mol. Biol..

[156] Hierro-Bujalance C., Bacskai B.J., Garcia-Alloza M. (2018). In vivo imaging of microglia with multiphoton microscopy. Front. Aging Neurosci..

[157] Neumann J., Henneberg S., Von Kenne S., Nolte N., Müller A.J., Schraven B., Görtler M.W., Reymann K.G., Gunzer M., Riek-Burchardt M. (2018). Beware the intruder: Real time observation of infiltrated neutrophils and neutrophil-Microglia interaction during stroke in vivo. PLoS ONE.

[158] Savage J.C., Carrier M., Tremblay M.È. (2019). Morphology of Microglia Across Contexts of Health and Disease. Methods Mol. Biol..

[159] Li Y., Du X.F., Liu C.S., Wen Z.L., Du J.L. (2012). Reciprocal Regulation between Resting Microglial Dynamics and Neuronal Activity In vivo. Dev. Cell.

[160] Wake H., Moorhouse A.J., Jinno S., Kohsaka S., Nabekura J. (2009). Resting microglia directly monitor the functional state of synapses in vivo and determine the fate of ischemic terminals. J. Neurosci..

[161] Chen Z., Ross J.L., Hambardzumyan D. (2019). Intravital 2-photon imaging reveals distinct morphology and infiltrative properties of glioblastoma-associated macrophages. Proc. Natl. Acad. Sci. USA.

[162] Bethge P., Chéreau R., Avignone E., Marsicano G., Nägerl U.V. (2013). Two-photon excitation STED microscopy in two colors in acute brain slices. Biophys. J..

[163] Evans T.A., Barkauskas D.S., Myers J.T., Huang A.Y. (2014). Intravital imaging of axonal interactions with microglia and macrophages in a mouse dorsal column crush injury. J. Vis. Exp..

[164] Fekete R., Cserép C., Lénárt N., Tóth K., Orsolits B., Martinecz B., Méhes E., Szabó B., Németh V., Gönci B. (2018). Microglia control the spread of neurotropic virus infection via P2Y12 signalling and recruit monocytes through P2Y12-independent mechanisms. Acta Neuropathol..

[165] Qiao S., Qian Y., Xu G., Luo Q., Zhang Z. (2019). Long-term characterization of activated microglia/macrophages facilitating the development of experimental brain metastasis through intravital microscopic imaging. J. Neuroinflamm..

[166] Tremblay M.Ě., Lowery R.L., Majewska A.K. (2010). Microglial interactions with synapses are modulated by visual experience. PLoS Biol..

[167] Heintzmann R., Huser T. (2017). Super-Resolution Structured Illumination Microscopy. Chem. Rev..

[168] Hahn C., Becker K., Saghafi S., Pende M., Avdibašić A., Foroughipour M., Heinz D.E., Wotjak C.T., Dodt H.U. (2019). High-resolution imaging of fluorescent whole mouse brains using stabilised organic media (sDISCO). J. Biophotonics.

[169] Qi Y., Yu T., Xu J., Wan P., Ma Y., Zhu J., Li Y., Gong H., Luo Q., Zhu D. (2019). FDISCO: Advanced solvent-based clearing method for imaging whole organs. Sci. Adv..

[170] Ormel P.R., de Sá R.V., van Bodegraven E.J., Karst H., Harschnitz O., Sneeboer M.A.M., Johansen L.E., van Dijk R.E., Scheefhals N., van Berlekom A.B. (2018). Microglia innately develop within cerebral organoids. Nat. Commun..

[171] Pfeiffer T., Poll S., Bancelin S., Angibaud J., Inavalli V.V.G.K., Keppler K., Mittag M., Fuhrmann M., Nägerl U.V. (2018). Chronic 2P-STED imaging reveals high turnover of dendritic spines in the hippocampus in vivo. eLife.

[172] Vangindertael J., Camacho R., Sempels W., Mizuno H., Dedecker P., Janssen K.P.F. (2018). An introduction to optical super-resolution microscopy for the adventurous biologist. Methods Appl. Fluoresc..

[173] Cserép C., Pósfai B., Lénárt N., Fekete R., László Z.I., Lele Z., Orsolits B., Molnár G., Heindl S., Schwarcz A.D. (2020). Microglia monitor and protect neuronal function via specialized somatic purinergic junctions. Science.

[174] Varga D.P., Menyhárt Á., Pósfai B., Császár E., Lénárt N., Cserép C., Orsolits B., Martinecz B., Szlepák T., Bari F. (2020). Microglia alter the threshold of spreading depolarization and related potassium uptake in the mouse brain. J. Cereb Blood Flow Metab..

[175] Fumagalli S., Fiordaliso F., Perego C., Corbelli A., Mariani A., De Paola M., De Simoni M.G. (2019). The phagocytic state of brain myeloid cells after ischemia revealed by superresolution structured illumination microscopy. J. Neuroinflamm..

[176] Schafer D.P., Lehrman E.K., Kautzman A.G., Koyama R., Mardinly A.R., Yamasaki R., Ransohoff R.M., Greenberg M.E., Barres B.A., Stevens B. (2012). Microglia Sculpt Postnatal Neural Circuits in an Activity and Complement-Dependent Manner. Neuron.

[177] Beckman D., Ott S., Donis-Cox K., Janssen W.G., Bliss-Moreau E., Rudebeck P.H., Baxter M.G., Morrison J.H. (2019). Oligomeric Aβ in the monkey brain impacts synaptic integrity and induces accelerated cortical aging. Proc. Natl. Acad. Sci. USA.

[178] Konishi H., Okamoto T., Hara Y., Komine O., Tamada H., Maeda M., Osako F., Kobayashi M., Nishiyama A., Kataoka Y. (2020). Astrocytic phagocytosis is a compensatory mechanism for microglial dysfunction. EMBO J..

[179] Weinhard L., Di Bartolomei G., Bolasco G., Machado P., Schieber N.L., Neniskyte U., Exiga M., Vadisiute A., Raggioli A., Schertel A. (2018). Microglia remodel synapses by presynaptic trogocytosis and spine head filopodia induction. Nat. Commun..

[180] Karreman M.A., Hyenne V., Schwab Y., Goetz J.G. (2016). Intravital Correlative Microscopy: Imaging Life at the Nanoscale. Trends Cell Biol..

[181] Van Ham T.J., Brady C.A., Kalicharan R.D., Oosterhof N., Kuipers J., Veenstra-Algra A., Sjollema K.A., Peterson R.T., Kampinga H.H., Giepmans B.N.G. (2014). Intravital correlated microscopy reveals differential macrophage and microglial dynamics during resolution of neuroinflammation. Dis. Model Mech..

[182] Luckner M., Burgold S., Filser S., Scheungrab M., Niyaz Y., Hummel E., Wanner G., Herms J. (2018). Label-free 3D-CLEM Using Endogenous Tissue Landmarks. Science.

[183] Cunningham C.L., Martínez-Cerdeño V., Noctor S.C. (2013). Microglia Regulate the Number of Neural Precursor Cells in the Developing Cerebral Cortex. J. Neurosci..

[184] Miyamoto A., Wake H., Moorhouse A.J., Nabekura J. (2013). Microglia and Synapse Interactions: Fine Tuning Neural Circuits and Candidate Molecules. Front. Cell Neurosci..

[185] Kettenmann H., Kirchhoff F., Verkhratsky A. (2013). Microglia: New Roles for the Synaptic Stripper. Neuron.

[186] Watson P.M.D., Kavanagh E., Allenby G., Vassey M. (2017). Bioengineered 3D Glial Cell Culture Systems and Applications for Neurodegeneration and Neuroinflammation. SLAS Discov..

[187] Pöttler M., Zierler S., Kerschbaum H.H. (2006). An Artificial Three-Dimensional Matrix Promotes Ramification in the Microglial Cell-Line, BV-2. Neurosci. Lett..

[188] Cho H.J., Verbridge S.S., Davalos R.V., Lee Y.W. (2018). Development of an In vitro 3D Brain Tissue Model Mimicking In vivo-Like Pro-Inflammatory and Pro-Oxidative Responses. Ann. Biomed. Eng..

[189] Haw R.T.Y., Tong C.K., Yew A., Lee H.C., Phillips J.B., Vidyadaran S. (2014). A Three-Dimensional Collagen Construct to Model Lipopolysaccharide-Induced Activation of BV2 Microglia. J. Neuroinflamm..

[190] Chronopoulou L., Togna A.R., Guarguaglini G., Masci G., Giammaruco F., Togna G.I., Palocci C. (2012). Self-Assembling Peptide Hydrogels Promote Microglial Cells Proliferation and NGF Production. Soft Matter.

[191] Park J., Wetzel I., Marriott I., Dréau D., D’Avanzo C., Kim D.Y., Tanzi R.E., Cho H. (2018). A 3D Human Tri-Culture System Modeling Neurodegeneration and Neuroinflammation in Alzheimer’s Disease. Nat. Neurosci..

[192] Cai H., Ao Z., Hu L., Moon Y., Wu Z., Lu H.C., Kim J., Guo F. (2020). Acoustofluidic Assembly of 3D Neurospheroids to Model Alzheimer’s Disease. Analyst.

[193] Lancaster M.A., Knoblich J.A. (2014). Organogenesis in a Dish: Modeling Development and Disease Using Organoid Technologies. Science.

[194] Lancaster M.A., Knoblich J.A. (2014). Generation of Cerebral Organoids from Human Pluripotent Stem Cells. Nat. Protoc..

[195] Dixit C.K., Kaushik A. (2016). Microfluidics for Biologists: Fundamentals and Applications.

[196] Park J.W., Vahidi B., Taylor A.M., Rhee S.W., Jeon N.L. (2006). Microfluidic Culture Platform for Neuroscience Research. Nat. Protoc..

[197] Gross P.G., Kartalov E.P., Scherer A., Weiner L.P. (2007). Applications of Microfluidics for Neuronal Studies. J. Neurol. Sci..

[198] Campenot R.B. (1977). Local Control of Neurite Development by Nerve Growth Factor. Proc. Natl. Acad. Sci. USA.

[199] Raff M.C., Whitmore A.V., Finn J.T. (2002). Axonal self-destruction and neurodegeneration. Science.

[200] Zweifel L.S., Kuruvilla R., Ginty D.D. (2005). Functions and Mechanisms of Retrograde Neurotrophin Signalling. Nat. Rev. Neurosci..

[201] Taylor A.M., Rhee S.W., Tu C.H., Cribbs D.H., Cotman C.W., Jeon N.L. (2003). Microfluidic Multicompartment Device for Neuroscience Research. Langmuir.

[202] Park J., Koito H., Li J., Han A. (2009). A Multi-Compartment CNS Neuron-Glia Co-Culture Microfluidic Platform. J. Vis. Exp..

[203] Rhee S.W., Taylor A.M., Tu C.H., Cribbs D.H., Cotman C.W., Jeon N.L. (2005). Patterned Cell Culture inside Microfluidic Devices. Lab. Chip..

[204] Taylor A.M., Blurton-Jones M., Rhee S.W., Cribbs D.H., Cotman C.W., Jeon N.L. (2005). A Microfluidic Culture Platform for CNS Axonal Injury, Regeneration and Transport. Nat. Methods..

[205] Hosmane S., Yang I.H., Ruffin A., Thakor N., Venkatesan A. (2010). Circular Compartmentalized Microfluidic Platform: Study of Axon-Glia Interactions. Lab Chip.

[206] Hosmane S., Tegenge M.A., Rajbhandari L., Uapinyoying P., Kumar N.G., Thakor N., Venkatesan A. (2012). Toll/Interleukin-1 Receptor Domain-Containing Adapter Inducing Interferon-β Mediates Microglial Phagocytosis of Degenerating Axons. J. Neurosci..

[207] Majumdar D., Gao Y., Li D., Webb D.J. (2011). Co-Culture of Neurons and Glia in a Novel Microfluidic Platform. J. Neurosci. Methods.

[208] Shi M., Majumdar D., Gao Y., Brewer B.M., Goodwin C.R., McLean J.A., Li D., Webb D.J. (2013). Glia Co-Culture with Neurons in Microfluidic Platforms Promotes the Formation and Stabilization of Synaptic Contacts. Lab. Chip..

[209] Fujita Y., Nakanishi T., Ueno M., Itohara S., Yamashita T. (2020). Netrin-G1 Regulates Microglial Accumulation along Axons and Supports the Survival of Layer V Neurons in the Postnatal Mouse Brain. Cell Rep..

[210] Fujita Y., Yamashita T. (2020). Protocol for Co-Culture of Microglia with Axons. STAR Protoc..

